# Induction of Triple-Negative Breast Cancer Cell Death and Chemosensitivity Using mTORC2-Directed RNAi Nanomedicine

**DOI:** 10.1158/2767-9764.CRC-24-0261

**Published:** 2025-03-19

**Authors:** Shrusti S. Patel, Rebecca S. Cook, Justin H. Lo, Fiona K. Cherry, Ella N. Hoogenboezem, Fang Yu, Nora Francini, Nina T. Cassidy, Joshua T. McCune, Eva F. Gbur, Lisa Messier, Thomas A. Dean, Kalin L. Wilson, Dana M. Brantley-Sieders, Craig L. Duvall

**Affiliations:** 1 Department of Biomedical Engineering, Vanderbilt University, Nashville, Tennessee.; 2 Division of Hematology/Oncology, Department of Medicine, Vanderbilt University Medical Center, Nashville, Tennessee.; 3 Department of Medicine, Vanderbilt University Medical Center, Nashville, Tennessee.

## Abstract

Alterations in the PI3K/mTOR signaling pathway are often seen in triple-negative breast cancers (TNBC), a breast cancer subtype characterized by limited molecularly targeted treatment options and poorer patient outcomes. We report that gene amplification or overexpression of the mTORC2-required cofactor *RICTOR* correlated with increased mTORC2 signaling and worse patient outcomes in clinical breast cancer expression datasets, supporting studies examining selective mTORC2 inhibition in TNBC. The mTOR kinase inhibitor PP242 blocks both mTORC1 and mTORC2, which decreases growth and survival of *RICTOR*-amplified TNBC cells. However, mTORC1 inhibition by PP242 causes resurgent PI3K signaling, limiting its therapeutic impact. In contrast, knockdown using siRNA designed against Rictor (siRictor) did not increase PI3K or mTORC1 signaling but potently blocked mTORC2 signaling, resulting in robust inhibition of tumor cell growth and survival. We developed siRictor-loaded nanoparticles to enable therapeutic testing of *RICTOR* silencing in TNBCs *in vivo*. Intravenous delivery of this mTORC2-selective nanomedicine achieved siRNA accumulation and on-target gene silencing in TNBC tumors *in vivo*. *RICTOR* silencing blocked tumor mTORC2 signaling and growth in multiple TNBC mouse models while also improving TNBC tumor response to chemotherapy. These findings support the further development of technologies for therapeutic *RICTOR* silencing as an effective approach for mTORC2-selective inhibition and treatment in TNBC.

**Significance:**

We identified an mTORC2/Rictor-directed RNAi nanomedicine that cooperates with chemotherapy to enhance *in vivo* tumor cell killing in PI3K-active TNBCs.

## Introduction

Triple-negative breast cancers (TNBC) comprise 15% to 20% of all breast cancers and lack key clinical biomarkers [estrogen receptor (ER), progesterone receptor (PR), and HER2] that guide established molecularly targeted treatments for other breast cancer subtypes. Intravenous infusion of cytotoxic chemotherapies, local radiation, and resection surgery are the most readily available options for patients with TNBC. However, up to 35% of patients with TNBC treated with chemotherapy will experience recurrence within 5 years of surgery (1, 2), and few molecularly targeted clinical therapies are available for recurrent TNBCs (3, 4). Mutations in the PI3K/mTOR/Akt pathway are found in up to 70% of all breast cancers (5–7). Notably, PI3K/mTOR signaling to Akt is a key oncogenic pathway driving tumor cell survival and treatment resistance (8, 9), and aberrant PI3K/mTOR pathway mutations are found in 50% of cases of residual disease (RD) in TNBCs following neoadjuvant chemotherapy (NAC; ref. 10). These findings support the increased interest in targeted inhibition of the PI3K/mTOR pathway in PI3K/mTOR-active TNBC, particularly in combination with chemotherapy.

The kinase mTOR functions in two distinct protein complexes, mTOR complex 1 (mTORC1) and mTOR complex 2 (mTORC2). Each complex has its own distinct regulatory roles that support cancer formation and progression (11, 12). mTORC1 is activated downstream of PI3K signaling, controlling anabolic cellular processes that support cell proliferation, including protein translation, through phosphorylation of eukaryotic translation initiation factor 4E–binding protein 1 (4EBP1) and ribosomal protein S6 kinase (S6K) effectors (Supplementary Schematic S1), while blocking catabolic processes, such as phago-endocytosis and autophagy (13). Given the correlation of mTORC1 signaling with anabolic growth and cell proliferation under conditions of sufficient nutrient availability, TNBC treatment with selective mTORC1 inhibitory rapalogues (everolimus/RAD001 and sirolimus) has been investigated, but they have not achieved clinical success, even when combined with chemotherapy (14, 15). Interestingly, mTORC1 signaling suppresses PI3K signaling in a negative feedback loop, such that mTORC1 inhibition releases this negative feedback, triggering resurgent PI3K signaling that activates mTORC2 and Akt (16), an mTORC2 substrate sitting at the apex of cell survival and treatment resistance pathways. Next-generation mTOR kinase inhibitors were expected to overcome these limitations, as these would block activity of both mTORC1 and mTORC2 (17–19). Although mTOR kinase inhibitors restrained mTORC2-mediated phosphorylation of Akt, they failed to block PI3K resurgence, resulting in partial Akt reactivation by the PI3K effector PDK1 (18, 19). Furthermore, mTOR kinase inhibitor treatment can lead to emergence of a drug resistant cancer stem cell–like population caused by mTORC1 inhibition (17). Although they face these obstacles, mTOR kinase inhibitors remain under clinical investigation for multiple cancer types (20–23).

Relatively less is known about the therapeutic benefits of mTORC2-specific targeting. This is due in part to an absence of small molecules that inhibit mTORC2 while sparing mTORC1 because the enzymatic subunit, mTOR, is common to both complexes. Selective mTORC2 inhibition might be possible using an approach targeting factors that distinguish mTORC2 from mTORC1. The mTORC2 cofactor Rictor is a defining feature of mTORC2 that is required for mTORC2 activity and is not a component of mTORC1. Interestingly, a growing body of evidence supports a role for Rictor/mTORC2 in breast cancer progression and therapeutic resistance. For example, Rictor staining intensity correlated with increased tumor grade in clinical datasets of invasive breast carcinomas. *RICTOR* alterations (gene amplification/mRNA overexpression) also correlated with decreased overall patient survival in invasive breast carcinoma datasets. In HER2-amplified breast cancer models, both *RICTOR* gene knockout and nanoparticle-based delivery of Rictor RNAi—an RNA-mediated strategy to block expression of a specific gene—blocked mTORC2/Akt-dependent tumor cell survival and metastasis *in vivo* (24) and improved tumor response to the HER2 kinase inhibitor lapatinib (25). In the context of TNBC, meta-analyses of clinical breast cancer expression datasets revealed that high *RICTOR* expression correlated with decreased progression-free survival in basal-like 1 and basal-like 2 TNBC subtypes (25).

In the current work, we assessed the impact of selective mTORC2 inhibition on TNBC cell growth using Rictor RNAi. We compared effects across *RICTOR*-amplified, *RICTOR*-diploid, and Rictor protein–overexpressing TNBC models, finding robust mTORC2 inhibition and tumor cell killing in PI3K-active TNBC. We formulated a Rictor-targeting siRNA nanomedicine for systemic delivery to TNBC-bearing mice, producing potent tumor Rictor knockdown, decreased tumor mTORC2 activity, substantial tumor growth inhibition, and increased tumor response to paclitaxel.

## Materials and Methods

This section describes the experimentation used to probe the central hypothesis that selective mTORC2 inhibition using siRNA-mediated Rictor ablation is a therapeutically beneficial strategy to inhibit tumor cell growth and survival and enhance chemosensitivity of *RICTOR*-amplified TNBC tumor cells.

### Materials

Unless noted, all chemicals and materials for biological assays were purchased from Sigma-Aldrich or Thermo Fisher Scientific. A list of oligonucleotide sequences is provided in Supplementary Table S2, and chemical modifications, if used, are indicated. Unmodified siRNAs were purchased from Dharmacon. siRNAs bearing modifications, including fluorophore labeling, were synthesized on a MerMade 12 Oligonucleotide Synthesizer (BioAutomation).

### Human breast cancer dataset and Cancer Cell Line Encyclopedia dataset analysis

To probe the clinical impact of *RICTOR* expression and genomic amplification in patients with breast cancer, clinical breast cancer datasets curated by The Cancer Genome Atlas (TCGA; ref. 26) and METABRIC (27, 28) were queried for tumors harboring *RICTOR* gene amplification or *RICTOR* mRNA overexpression (defined as >2 SD above the mean *RICTOR* levels across the entire dataset), collectively defined herein as *RICTOR* alterations, using cBioPortal software (RRID: SCR_014555; ref. 29). Kaplan–Meier survival curves were generated using cBioPortal software comparing overall survival (OS) of patients whose tumors harbor *RICTOR* alterations versus those without *RICTOR* alterations. Reverse phase protein array (RPPA) datasets of TCGA-curated invasive breast cancer were analyzed for Rictor protein overexpression and Rictor protein phosphorylation levels using cBioPortal software to determine whether Rictor protein overexpression correlates with patient survival and phosphorylation of mTORC2 substrates. RPPA analysis of TCGA breast invasive carcinoma was assessed by KmPlot software (RRID: SCR_018753), using software-directed selection of expression cutoff criteria. Clinical parameters embedded in datasets were used in KmPlot software to assess the correlation between Rictor protein phosphorylation analysis and OS within breast cancer clinical subtypes (ER^+^/HER2^−^, ER^+^/HER2^+^, ER^−^/HER2^−^, and ER^−^/HER2^+^).

To identify TNBC cell lines harboring PI3K/mTOR genomic alterations, cell lines curated by the Cancer Cell Line Encyclopedia (CCLE; ref. 30) were queried in cBioPortal software for TNBC characterization (defined as lacking *ESR1* expression >1 SD above the groups average and/or *ERBB2* gene amplification). The genomic copy number for selected gene data was analyzed for all cell lines.

### Cell culture and treatment

Human TNBC cell lines HCC70 (cat #CRL-2315, RRID: CVCL_1270), HCC1937 (cat #CRL-2336, RRID: CVCL_0290), BT20 (cat #HTB-19, RRID: CVCL_0178), MDA-MB-157 (cat #HTB-24, RRID: CVCL_0618), BT549 (cat #HTB-122, RRID: CVCL_1092), MDA-MB-231 (cat #HTB-26, RRID: CVCL_0062), MDA-MB-436 (HTB-130, RRID: CVCL_0623), HCC1599 (cat #CRL-2331, RRID: CVCL_1256), and HCC1187 (cat #CRL-2322, RRID: CVCL_1247) were purchased from the ATCC. CAL-85-1 (cat #ACC 440, RRID: CVCL_1114) and CAL-120 (cat #ACC 459, RRID: CVCL_1104) were purchased from DSMZ. Cell line re-authentication was not conducted following receipt of cells from the cell banks. Cell lines were passaged at least once after thawing from cryostorage prior to use in experiments and maintained at <50 passages for all experiments. Total culture time for cells did not exceed 3 months. All cell lines were screened regularly every 6 months for *Mycoplasma* using the MycoAlert Mycoplasma Detection Kit (Lonza). All cells were maintained in 10% FBS (Thermo Fisher Scientific) and 1% antibiotic–antimycotic containing penicillin, streptomycin, and amphotericin B (Anti-Anti, Thermo Fisher Scientific) using RPMI-1640 media (Thermo Fisher Scientific) for HCC70, HCC1187, HCC1937, and HCC1599 and DMEM (Thermo Fisher Scientific) for CAL-85-1, CAL-120, MDA-MB-157, MDA-MB-436, BT20, BT549, and MDA-MB-231.

All small-molecule drugs, apart from siRNAs, for cell treatment were purchased from SelleckChem. Where indicated, cells were treated with RAD001 (200 nmol/L), PP242 (500 nmol/L), paclitaxel (dose range from 0–500 nmol/L), or doxorubicin (0–10 μmol/L) dissolved in DMSO; an equal volume of DMSO was used as a vehicle control. For dose range experiments, the cells were treated with a twofold dilution of the given drug. Where indicated, the cells were reverse-transfected with 100 nmol/L siRNA complexed with Lipofectamine RNAiMAX (Thermo Fisher Scientific) in Opti-MEM media (Thermo Fisher Scientific) according to the manufacturer’s instructions. Where indicated, adhered cells were treated with 100 nmol/L siRNA formulated into our custom siRNA nanoparticles (si-NP) in Opti-MEM, and treatment media were replaced with complete media after 24 hours.

### Western blotting

Cells were assessed by Western blot assays to identify signaling effects that resulted from treatment with our various drugs and siRNAs. Cells cultured in six-well plates were lysed in RIPA buffer supplemented with a protease inhibitor (cOmplete, Roche) and phosphatase inhibitor (PhosSTOP, Roche) for 15 minutes on ice. The lysates were then cleared by centrifugation (13,000 × *g* for 10 minutes at 4°C). Tumors were homogenized using the TissueLyser LT (Qiagen) and then incubated in RIPA buffer on ice for ≥1 hour. Protein concentration was quantified by Pierce BCA assay (Thermo Fisher Scientific). Proteins (30 μg) were resolved on 4% to 12% polyacrylamide gels (Novex, Invitrogen) and transferred to nitrocellulose membranes (iBlot2, Invitrogen). The membranes were blocked and probed with the following mouse monoclonal antibodies obtained from Santa Cruz Biotechnology: actin (1:5,000, cat #sc-130065, RRID: AB_1249316), P-4EBP1 (1:2,000, cat #sc-293124, RRID: AB_2943677), and S6 (1:1,000, cat #sc-74459, RRID: AB_1129205); the following rabbit monoclonal antibodies were purchased from Cell Signaling Technology: Raptor (1:500, cat #2280, RRID: AB_561245), Rictor (1:500, cat #2114, RRID: AB_2179963), mTOR (1:1,000, cat #2983, RRID: AB_2105622), total Akt (1:2,000, cat #4691, RRID: AB_915783), phosphorylated Akt (P-Akt) S473 (1:2,000, cat #4060, RRID: AB_2315049), P-Akt T308 (1:1,000, cat #4056, RRID: AB_331163), P-S6 (1:2,000, cat #4858, RRID: AB_916156), p70 S6K (1:2,000, cat #34475, RRID: AB_2943679), P-p70 S6K (1:2,000, cat #97596, RRID: AB_2800283), 4EBP1 (1:3,000, cat #9644, RRID: AB_2097841), protein kinase Cα (PKCα, 1:2,000, cat #59754, RRID: AB_2799573), P-PKCα (1:1,000, cat #9375, RRID: AB_2284224), GSK3β (1:1,000, cat #12456, RRID: AB_2636978), and P-GSK3β (1:1,000, cat #5558, RRID: AB_10013750). The membranes were probed with anti–mouse IgG IRDyes (1:15,000, cat #926-32210, RRID: AB_621842) or anti–rabbit IgG IRDyes (1:15,000, cat #926-32211, RRID: AB_621843, 1:15,000) obtained from LI-COR Biosciences. Gels were imaged using the Odyssey Fc Imaging System (LI-COR Biosciences).

### Cell number and caspase 3/7 activity

Following treatment, cells were assessed for cell number and caspase 3/7 activity to quantify the effect of various drugs on cell growth and cell death. Caspase 3/7 activity was selected to measure cell apoptosis because this molecular event is an early indicator of irreversible cell death. Cells were seeded in opaque 96-well plates and were then treated with RAD001 or PP242 ± paclitaxel or doxorubicin at indicated doses. The cells were reverse-transfected with siRNA non-targeting control (siControl), siRNA Raptor (siRaptor), or siRNA Rictor (siRictor) at the indicated doses, replacing the transfection media after 24 hours. Caspase 3/7 activity was assessed at 48 hours, unless otherwise stated, by Caspase-Glo 3/7 assay (Promega). The cell number was assessed at 72 hours, unless otherwise stated, by the CellTiter-Glo assay (Promega). Seeded cells were treated with siControl-NPs or siRictor-NPs at indicated doses in Opti-MEM, replacing treatment media with full serum media at 24 hours. The cell number was assessed at 2 or 7 days, unless otherwise stated, after treatment. Luminescence for each assay was measured on a Tecan Infinite M1000 plate reader. Data were normalized to the average value of untreated control wells.

For *in vitro* synergy experiments, HCC70 cells were seeded in black-walled 96-well plates treated by reverse transfection with siRictor at the doses of 0, 1, 3.16, 10, or 31.6 nmol/L using Lipofectamine RNAiMAX (Thermo Fisher Scientific). After 48 hours, treatment media were aspirated and replaced with full-serum media containing paclitaxel (0, 1, 3.16, 10, or 31.6 nmol/L) or doxorubicin (0, 3.16, 10, 31.6, 100, or 316 nmol/L). Paclitaxel treatment was maintained for 48 hours, for a total treatment duration of 96 hours, whereas doxorubicin treatment was maintained for 72 hours, for a total treatment of 120 hours. Cell viability was then assessed by CellTiter-Glo (Promega). CellTiter-Glo luminescence was normalized to untreated controls. Synergy scores were computed and visualized using SynergyFinder 3.0 (31).

### qRT-PCR

Following siRNA treatment, qRT-PCR assays were used to quantify the knockdown of targeted genes. mRNA was isolated from cells cultured in six-well plates using the RNeasy Mini Kit (Qiagen) and then reverse-transcribed using the iScript cDNA Synthesis Kit (Bio-Rad) following the manufacturer’s instructions. Relative mRNA was quantified using SYBR Green (Bio-Rad) qRT-PCR on a Bio-Rad CFX96 real-time PCR machine, normalizing mRNA to the housekeeping gene *GAPDH* using primers listed in Supplementary Table S3.

### Phospho-array assay and measurement of phosphatidyl inositol (3,4,5)-phosphate

Following PP242 and siRNA treatment, phospho-array assays were used to quantify kinase signaling effects. Cells were transfected in Opti-MEM with 100 nmol/L siControl and siRictor sequences, replacing transfection media with Opti-MEM 16 hours after transfection. Adhered cells were treated with 500 nmol/L PP242. After 2 or 7 days, cell lysates were collected in NP-40 lysis buffer supplemented with EDTA-free protease inhibitor cocktail (Roche) and PhosSTOP (Roche), cleared by centrifugation at 14,000 × *g* for 10 minutes at 4°C, and assessed for protein concentration using Pierce BCA assay (Thermo Fisher Scientific). Protein (400 μg) per membrane was used according to the manufacturer’s instructions in the Proteome Profiler Human Phospho-Kinase Array kit (R&D Systems). The membranes were probed with goat anti-rabbit antibody (cat #926-32211, RRID: AB_621843, 1:15,000) from LI-COR Biosciences and imaged on LI-COR Odyssey. Densitometry analysis was performed using Fiji (ImageJ, RRID: SCR_002285).

Following PP242 and siRNA treatment, cells were quantified for phosphatidyl inositol (3,4,5)-phosphate [PI(3,4,5)P3] levels as a measure of PI3K signaling. Cells were transfected with Lipofectamine in Opti-MEM with 100 nmol/L siControl and siRictor or without Lipofectamine using 200 nmol/L siControl-NP or siRictor-NP, replacing transfection media with fresh Opti-MEM 16 hours after transfection. Adhered cells were treated with 500 nmol/L PP242. After 48 hours, cell lysates were collected in RIPA lysis buffer supplemented with EDTA-free protease inhibitor cocktail (Roche) and PhosSTOP (Roche). Protein concentration was quantitated by Pierce BCA assay (Thermo Fisher Scientific). Lysates were used as directed in the PI3-Kinase Activity ELISA: Pico kit (Echelon Biosciences Inc.), which measures the PI3K-mediated conversion of PI(4,5)P2 to PI(3,4,5)P3. Kinase reactions were supplemented with 2 mmol/L dithiothreitol and 100 μmol/L ATP and incubated at 37°C for 6 hours prior to use in ELISA. The absorbance of ELISA reactions was measured at 450 nm on the Tecan Infinite M1000 plate reader. PI(3,4,5)P3 values for each kinase reaction were calculated against a PI(3,4,5)P3 standard curve. For siRictor-NP experiments, adhered cells were treated with either 100 nmol/L si-NPs or 250 nmol/L PP242, and lysates were collected at 72 hours.

### Flow cytometry

Flow cytometry was used to quantify cell proliferation, apoptosis, and cell-cycle stage following si-NP treatment. Cells were treated with 200 nmol/L siControl-NP and siRictor-NP treatments in serum-free DMEM for 16 hours and then cultured in full-serum media for 72 hours, adding bromodeoxyuridine (BrdU, 10 μg/mL) for the final 24 hours of culture. For annexin V analysis, cells were collected by trypsinization, resuspended in Annexin V Binding buffer (Invitrogen), and stained with annexin V-488 and propidium iodide (PI) using the Dead Cell Apoptosis Kit for flow cytometry (Invitrogen) according to the manufacturer’s instructions. Apoptotic cells were defined as annexin V-positive and PI-negative. For BrdU incorporation assays and annexin V-staining assays, trypsinized cells were collected, washed, then fixed with ice-cold 75% methanol, and permeabilized with 0.1% Triton X-100; 1 × 10^6^ cells were used for staining. For BrdU detection, DNA was denatured using 1 mol/L HCl for 10 minutes, the cells were washed in PBS and blocked in cell staining buffer supplemented with 5% normal mouse serum, and then incubated in mouse monoclonal anti-BrdU FITC (Invitrogen, cat #11-5071-42, RRID: AB_11042627, 1:100). For cell-cycle analysis, the cells were stained with PI/RNaseA solution using the Propidium Iodide Flow Cytometry Kit for cell-cycle analysis (Abcam) as directed by the manufacturer’s protocol. The cells were analyzed on a Guava easyCyte HT flow cytometer (Luminex). Flow cytometry data were analyzed on FlowJo software (RRID: SCR_008520) by comparing against unstained and single-stained controls. G_1_, S, or G_2_–M-phases were distinguished using cell cycle modeling in FlowJo software.

### Polymer synthesis and si-NP formulation

Polymer synthesis and formulation of our si-NP to enable robust *in vivo* delivery of siRictor was performed as previously described (32). Briefly, polymers were synthesized by reversible addition-fragmentation chain transfer (RAFT) polymerization using 4-cyano-4-(ethylsulfanylthiocarbonyl)sulfanylpentanoic acid (ECT) as the chain transfer agent (CTA). 50B polymers were synthesized at a 50:50 molar ratio of 2-(dimethylamino)ethyl methacrylate (DMAEMA) and butyl methacrylate (BMA). AIBN was used as the radical initiator at a 5:1 ratio of CTA:initiator in dioxane, and the polymerizations were run for 24 hours. 50B was purified by precipitation in pentane. To synthesize 20k polyethylene glycol (PEG)-50B diblock copolymer, 20kPEG was first conjugated to ECT by diisopropylcarbodiimide/4-(N,N-dimethylamino)pyridine (DIC/DMAP) coupling of hydroxyl-terminated 20 kDa PEG to ECT in dichloromethane for 48 hours. A 10× molar excess of ECT, 10× DIC, and 5× DMAP to 20 kDa PEG was used in the reaction. 20kPEG-ECT was purified by precipitation in diethyl ether. RAFT polymerization of 50:50 DMAEMA and BMA from 20kPEG-ECT was performed as described above. 20kPEG-50B was purified by precipitation in 2:1 pentane:diethyl ether.

si-NPs were formulated using a 3 mg/mL polymer solution of 20kPEG-50B and 0.5 mg/mL polymer solution of 50B made up of 10% ethanol and 90% 0.1 mol/L citrate buffer (pH 4). si-NPs were formulated at a total polymer to nucleic acid ratio (calculated using the ratio of protonated amines on the polymer to phosphates on the siRNA duplex) of 16. Calculated amounts of polymer and siRNA were mixed, and the solution was allowed to complex for 30 minutes. To bring the solution to physiologic pH, 5× v/v 0.2 mol/L phosphate buffer (pH 8) was then added. si-NPs were concentrated using 50 kDa Amicon centrifugal tubes (Millipore Sigma). Afterward, 8× v/v of 300 mmol/L trehalose solution was added to concentrated si-NPs, and si-NPs were flash-frozen, lyophilized overnight, and stored at −80°C. To reconstitute lyophilized si-NPs, water was first added to the si-NP cake at the volume that si-NPs were lyophilized in. si-NPs were allowed to sit for 30 minutes to rehydrate fully, and si-NPs were diluted to their final working dose using media. For *in vivo* experiments, si-NPs were concentrated to a 1 mg/kg dose in a 100 μL injection volume containing 300 mmol/L trehalose, frozen, and lyophilized. *In vivo* lyophilized si-NPs were reconstituted with 100 μL sterile water to deliver si-NPs in an isotonic trehalose solution.

### Flow cytometry, organ biodistribution, and fluorescence imaging for si-NP uptake into TNBC xenografts

Flow cytometry and biodistribution assays were used to quantify the delivery of si-NPs to the tumor following intravenous treatment. Athymic (Foxn1^nu/nu^) female mice (4–6 weeks of age, RRID: IMSR_ENV:HSD-069) purchased from Envigo were injected with 1 × 10^6^ HCC70 cells in 100 μL Matrigel (Corning) in the left inguinal mammary fat pad. Once tumors reached a volume of 100 mm^3^, mice were intravenously injected with either a 300 mmol/L trehalose vehicle control or 1 mg/kg weight si-NPs harboring Cy5-tagged siRNAs. Organs and tumors were harvested 24 hours after treatment and imaged for Cy5 fluorescence on an IVIS System (Caliper Life Sciences). Baseline autofluorescence of each organ was normalized to vehicle-treated organs. Tumor uptake of Cy5-tagged si-NPs was measured in tumors digested with the Mouse Tumor Dissociation Kit (Miltenyi Biotec) following the manufacturer’s instructions. These samples were strained through a 70-μmeter filter, and red blood cells were lysed for 2 minutes at room temperature using ACK Lysis solution (Thermo Fisher Scientific). The cells were resuspended in FACS buffer (PBS containing 1% FBS and 2 mmol/L EDTA) to a concentration of 5 × 10^6^ cells per mL. A total of 1 × 10^6^ cells were incubated with 4′,6-diamidino-2-phenylindole (DAPI) and analyzed on a Guava easyCyte HT flow cytometer (Luminex). Flow cytometry data were analyzed on FlowJo software (RRID: SCR_008520), in which Cy5 uptake was quantified against vehicle-treated tumors. To visualize Cy5 signal within the tumor, tumor samples were snap-frozen in an optimal cutting temperature (OCT) embedding medium. Cryosections were stained with DAPI and imaged on a Nikon Eclipse Ti inverted confocal microscope.

### siRictor-NP monotherapy or combination chemotherapy in TNBC xenografts

TNBC xenograft studies were performed to assess the therapeutic impact of siRictor-NPs in preclinical breast cancer models. Athymic nude mice (4–6 weeks of age, RRID: IMSR_ENV:HSD-069) purchased from Envigo were injected with 1 × 10^6^ HCC70, CAL-85-1, or HCC1937 cells in 100 μL Matrigel in their left inguinal mammary fat pad. Once tumors reached a volume of 100 mm^3^ (day 0), mice were randomized into treatment groups.

The siRictor-NP monotherapy study was performed in HCC70, CAL-85-1, and HCC1937 tumor–bearing mice to assess the acute effects of siRictor-NP therapy and the impact of siRictor-NP therapy on mTORC2 signaling in the tumor. Mice were randomized into siControl-NP and siRictor-NP groups. Mice were treated intravenously with 1 mg/kg si-NPs on days 0, 2, and 4. Tumor volume was monitored using digital caliper measurements (T_vol_ = length × width^2^/2). Tumors were harvested at the study endpoint for molecular analysis. CAL-85-1 tumor–bearing mice were treated on days 0, 3, and 7 and weekly thereafter until the study endpoint at day 28.

The siRictor-NP combination chemotherapy study was performed to assess the long-term therapeutic effects of siRictor-NP therapy and their potential to cooperate with chemotherapy treatment. Mice were randomized into siControl-NPs + vehicle, siRictor-NPs + vehicle, siControl-NPs + paclitaxel, and siRictor-NPs + paclitaxel groups. Mice were treated intravenously with 1 mg/kg weight si-NPs thrice in the first week (days 0, 3, and 7) and weekly thereafter. Mice were treated intraperitoneally with 12 mg/kg weight paclitaxel twice a week, starting on day 2 and then once weekly starting on day 44. Tumor volume was monitored using digital caliper measurements (T_vol_ = length × width^2^/2). Tumors were harvested at the study endpoint for molecular analysis.

### Histologic analysis

Following the study endpoints, tumors were histologically assessed to identify the molecular impact of siRictor-NPs and chemotherapy. Tumors were harvested and fixed in 10% neutral-buffered formalin. Tissue processing and embedding was performed by the Vanderbilt Translation Pathology Shared Resource. Paraffin sections (4 μmeterol/L) were stained with hematoxylin (Thermo Fisher Scientific) and eosin (Abcam). IHC was performed using antibodies against Ki67 (Abcam, cat #ab16667, RRID: AB_302459, 1:500), cleaved caspase-3 (Cell Signaling Technology, cat #9664, RRID: AB_2070042, 1:100), and P-Histone H3 Ser10 (Cell Signaling Technology, cat #9701, RRID: AB_331535, 1:100). Primary antibody binding was visualized using VECTASTAIN Elite ABC-HRP Kit, Peroxidase (Rabbit IgG; cat #PK-6101, RRID: AB_2336820) from Vector Laboratories or Rabbit specific EnVision+ HRP Micropolymer Detection Kit (cat #K4003, RRID: AB_2630375) from Agilent. Images were captured using a Nikon Czsi+ confocal microscopy system.

### Ethics statement

All animals were housed under pathogen-free conditions, and experiments were performed in accordance with Association for Assessment and Accreditation of Laboratory Animal Care International guidelines and with Vanderbilt University Institutional Animal Care and Use Committee approval (protocol #M1600094).

### Statistical analysis

Data were analyzed using GraphPad Prism 10 software (RRID: SCR_002798). To provide the greatest level of detail, specific statistical tests used for data are indicated in corresponding captions. In general, a treatment group was compared with a control group using an unpaired *t* test. Multiple groups were compared to a control or to each other using one-way ANOVA with a Tukey multiple comparison *post hoc* test. For all figures, *, *P* ≤ 0.05; **, *P* ≤ 0.01; ***, *P* ≤ 0.001; ****, *P* ≤ 0.0001; and ns denotes not significant.

### Data availability

The data generated by the authors in this work are available upon request from the corresponding authors.

## Results

### *RICTOR* overexpression in clinical breast cancers predicts increased mTORC2 signaling and correlates with a worse outcome

**Figure 1:**
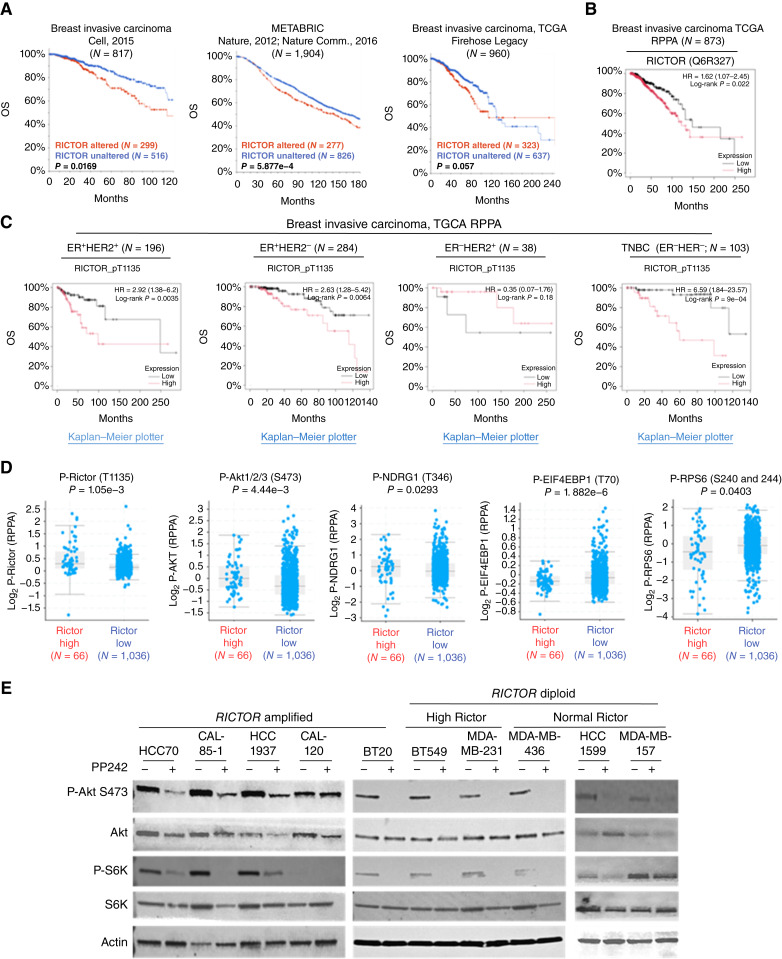
Rictor alterations correlate with increased mTORC2 signaling and decreased survival in invasive breast cancers. **A–C,** Kaplan–Meier curves illustrating the impact of (**A**) *RICTOR* alterations (gene amplification and/or mRNA expression >2 SDs above the dataset average) on OS in invasive breast cancer in three clinical gene expression datasets using cBioPortal software; (**B**) Rictor protein expression (software-defined best cutoff) on OS in TCGA invasive breast cancer RPPA datasets using KmPlot software; (**C**) Rictor phosphorylation at T1135 (software-defined best cutoff) on OS in TCGA invasive breast cancer RPPA datasets using KmPlot software. **D,** Invasive breast cancers (TCGA) were separated in *RICTOR* high (those with *RICTOR* gene amplification and/or mRNA expression >2 SD above the dataset average) or *RICTOR* low (all remaining tumors). Then RPPA data were assessed for phosphorylation of mTORC2 effectors Rictor (T1135), AKT (S473), and NDRG1 (T346) and for mTORC1 effectors EIF4EBP1 (T70) and RPS6 (S240/244). Each point represents a unique tumor sample. Midline is dataset average ± SD. An unpaired *t* test was used. **E,** Western blot analysis of serum-starved *RICTOR*-amplified and *RICTOR*-diploid cell lines treated with PP242 for 24 hours, then stimulated 30 minutes with insulin (10 ng/mL) and EGF (2 ng/mL). Antibodies used are indicated on the left.

To understand the clinical relevance of Rictor/mTORC2 in malignant breast cancers, we assessed *RICTOR* gene alterations in publicly available datasets curated by TCGA (Cell, *N* = 817; ref. 26), METABRIC (*N* = 1904; ref. 27), and TCGA Firehose Legacy (*N* = 960; refs. 29, 33), finding *RICTOR* gene amplification or overexpression in 36.6% (*N* = 299), 14.5% (*N* = 277), and 33.6% (*N* = 323) of tumor samples, respectively (Fig. 1A). In each dataset, tumor *RICTOR* amplification/overexpression correlated with a decreased patient OS, consistent with previous reports correlating *RICTOR* mRNA expression with decreased survival in patients with breast cancer (25). Similarly, high Rictor protein expression assessed by RPPA correlated inversely with OS in invasive breast cancers (Fig. 1B), as did Rictor phosphorylation at T1135 (P-Rictor T1135; Supplementary Fig. S1A), a phospho-site correlating with, but not required for, oncogenic mTORC2 signaling (34). KmPlot software was used to assess Rictor T1135 phosphorylation within TCGA invasive breast tumors using RPPA data, further separating the tumors into groups based on clinical assessments of ER and HER2 expression: ER^+^/HER2^+^ (*N* = 196), ER^+^/HER2^−^ (*N* = 284), ER^−^/HER2^+^ (*N* = 38), and TNBC:ER^−^HER2^−^ (*N* = 103). P-Rictor T1135 correlated with decreased OS in three of four subgroups, with the greatest impact seen in TNBC:ER^−^HER2^−^ tumors (Fig. 1C). P-Rictor T1135 did not significantly correlate with decreased OS in the ER^−^/HER2^+^ group. This could be due to the smaller population size of this group or may indicate that mTORC2 signaling does not affect survival in this clinical breast cancer subset. As Rictor is a key component of mTORC2, we assessed the RPPA dataset of invasive breast cancers (TCGA) to determine whether *RICTOR* gene expression or amplification correlates with increased mTORC2 signaling in clinical tumor specimens. Tumors with high *RICTOR* alteration harbored significantly higher P-Rictor T1135, P-Akt S473, and P-NDRG1, consistent with increased mTORC2 signaling in *RICTOR*-altered tumors (Fig. 1D). In contrast, mTORC1 effectors P-EIF4EBP1 and P-RPS6 exhibited diminished phosphorylation in *RICTOR*-altered breast tumors.

To identify relevant TNBC cell lines to use as models of *RICTOR*-amplified TNBC, we assessed genomic copy-number variations within 71 breast-derived cell lines profiled in the CCLE. After excluding breast lines with ER (*ESR1*) expression and HER2 (*ERBB2*) gene amplification, 14 of 35 remaining lines displayed *RICTOR* amplification (10/35) or protein overexpression (4/35; Supplementary Fig. S1B). Interestingly, 90% of *RICTOR* amplifications co-occurred with PI3K pathway alterations (either *PTEN* loss/mutation or *PIK3CA* amplification). From this analysis, we selected five *RICTOR*-amplified (HCC70, CAL-85-1, HCC1937, CAL-120, and BT20) lines for further analysis (Supplementary Table S1). Similarly, five *RICTOR*-diploid TNBC lines were selected and further partitioned by high Rictor protein levels (BT549 and MDA-MB-231) or normal Rictor protein levels (MDA-MB-436, HCC1599, and MDA-MB-157). The treatment of cell lines with the mTOR kinase inhibitor PP242 decreased mTORC2 signaling, as measured by decreased P-Akt S473, as well as mTORC1 signaling, measured by P-p70 S6 kinase (Fig. 1E). This result suggests that an intact mTORC2 signaling pathway is present in each cell line.

### mTOR kinase inhibition decreases TNBC cell growth but re-activates PI3K signaling

**Figure 2:**
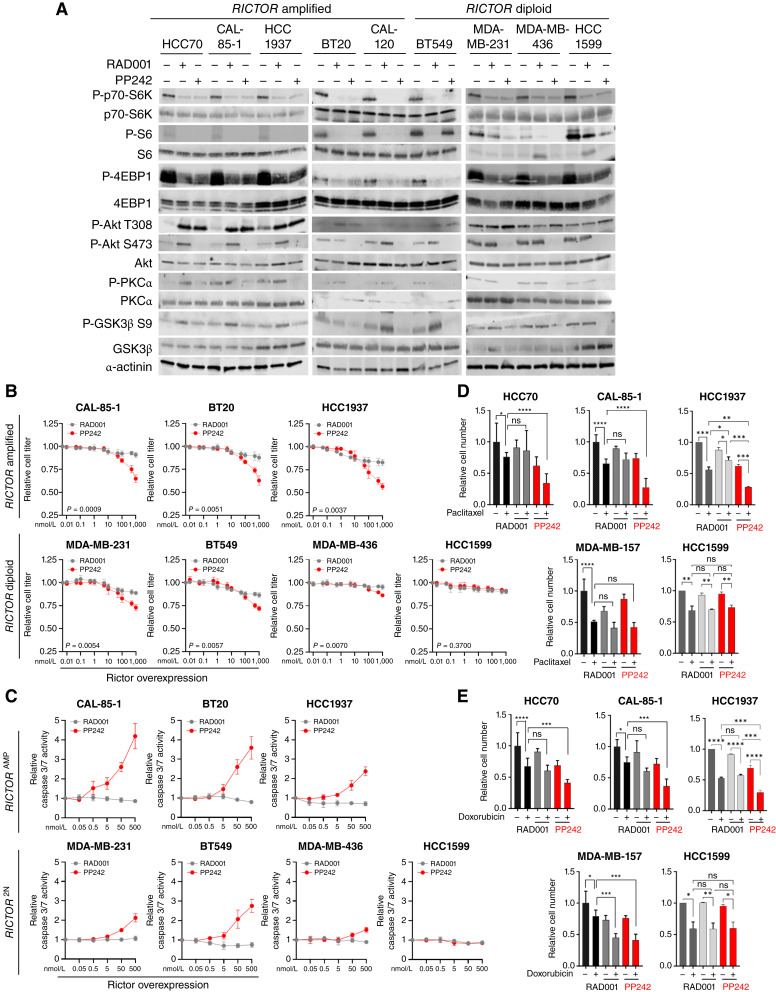
Combined mTORC1/2 inhibition is more effective than selective mTORC1 inhibition in TNBC. **A,** The distinct effects of mTORC1 inhibition in TNBC were studied using RAD001, whereas combined effects of dual mTORC1/2 inhibition were studied using mTOR kinase inhibitor PP242. Western blot analysis of whole-cell lysates harvested 24 hours after treatment with DMSO, RAD001 (500 nmol/L), or PP242 (500 nmol/L) in 10% serum. Antibodies used are indicated to the left of each panel. **B** and **C,** Cell titer (**B**) and caspase-(3/7) activity (**C**) were assessed as a measure of total cell number and apoptosis, respectively, in TNBC cells treated with increasing concentrations of RAD001 and PP242. Values shown are the average ± SD of *N* = 3, each assessed in duplicate. *P* values calculated using an unpaired *t* test of AUC analysis. **D** and **E,** Cell titer was assessed in TNBC cells treated with paclitaxel (**D**) or doxorubicin (**E**) alone or in combination with RAD001 (200 nmol/L) or PP242 (250 nmol/L). Values shown are the average ± SD of *N* = 2–3 biological replicates, each assessed in triplicate. *P* values calculated using one-way ANOVA.

To determine the distinct impacts of mTORC1 versus mTORC1/2 inhibition, we used the selective mTORC1 inhibitor everolimus/RAD001 and the mTOR1/2 kinase inhibitor PP242 (Fig. 2A; ref. 35). As expected, RAD001 blocked phosphorylation of mTORC1 effectors p70 S6 kinase, 4EBP1, and S6 but did not inhibit phosphorylation of the mTORC2 substrates Akt and PKCα or the Akt substrate GSK-3β. Instead, four of the five *RICTOR*-amplified cell lines showed increased P-Akt at T308, the PDK1 phosphorylation site, and at S473, the mTORC2 phosphorylation site, upon RAD001 treatment. RAD001 treatment-induced P-Akt upregulation was seen in only one of the five *RICTOR*-diploid cell lines tested. Given that mTORC2 and PDK1 are both activated in response to PI3K, this finding is consistent with previous reports showing increased PI3K signaling in response to mTORC1 inhibition (16). Decreased mTORC1 effector phosphorylation was also seen in cells treated with PP242, the dual mTORC1/2 kinase inhibitor, as was S473 phosphorylation of the mTORC2 substrate Akt. However, PP242 increased P-Akt T308 in four of the five *RICTOR*-amplified cell lines (HCC70, CAL-85-1, HCC1937, and BT20). These effects were studied in greater detail using Western blot analysis to assess the dynamic molecular response to mTORC1 versus mTORC1/mTORC2 blockade (Supplementary Fig. S2), revealing P-Akt S473 upregulation within 8 hours of mTOR inhibition. These findings suggest that mTOR kinase inhibition blocks both mTORC1 and mTORC2 signaling, but at the cost of increased PI3K signaling due to lost mTORC1 signaling.

In *RICTOR*-amplified cells cultured in full growth media, PP242 decreased cell growth in a dose-dependent manner (Fig. 2B). Similar results were seen in TNBC cells lacking *RICTOR* amplification but expressing high Rictor protein (MDA-MB-231 and BT549), whereas *RICTOR*-diploid cells with normal Rictor protein expression (MDA-MB-436 and HCC1599) displayed minimal sensitivity to dual mTORC1/2 inhibition. Although mTOR kinase inhibitors block signaling through mTORC2, the simultaneous inhibition of mTORC1 must also be considered. This is particularly relevant in the context of PI3K active cancers, given that loss of mTORC1-mediated restraint on the PI3K pathway may be a disadvantage (16). Although RAD001 produced dose-dependent growth inhibition in *RICTOR*-amplified cells and cells with high Rictor protein expression, the effect was more moderate than the impact of treatment with PP242, suggesting that dual mTORC1/mTORC2 blockade is more efficacious than mTORC1 blockade alone (Fig. 2B).

Given that mTORC2 is a key driver of cancer cell survival (36, 37), we assessed apoptosis in cells treated with PP242 by measuring the activity of caspase 3/7, a key molecular switch in the intrinsic apoptotic cascade. mTOR signaling is a major regulator of several cell death processes including apoptosis, necrosis, ferroptosis, and others (38); in this work, we measured caspase 3/7 activity as an early indicator of irreversible cell death. PP242 increased caspase 3/7 activity up to fourfold in *RICTOR*-amplified cells and over twofold in Rictor-high cells (Fig. 2C). PP242 had little to no induction of caspase 3/7 activity in *RICTOR*-diploid cells that were also normal for Rictor protein expression. In contrast, RAD001 did not increase caspase 3/7 activity in any cell line, suggesting that mTORC1 inhibition alone is not an effective activator of apoptosis in these cells, unlike mTORC1/mTORC2 combined inhibition.

Chemotherapy remains a standard of care for patients with TNBC. Because a substantial fraction of chemotherapy-resistant TNBC tumors harbor PI3K pathway alterations (10), we compared the impact of RAD001 and PP242 treatment on chemotherapy-mediated growth inhibition in *RICTOR*-amplified cells. Cells treated with paclitaxel or doxorubicin showed a dose-dependent decrease in cell viability (Supplementary Fig. S3A). In *RICTOR*-amplified HCC70, CAL-85-1, and HCC1937 cells, the combination of PP242 with paclitaxel (Fig. 2D) or PP242 with doxorubicin (Fig. 2E) resulted in greater growth inhibition compared with either agent alone, whereas the combination of RAD001 with either paclitaxel or doxorubicin did not. PP242 did not affect paclitaxel-mediated growth inhibition in *RICTOR*-diploid MDA-MB-157, HCC1599, and MDA-MB-436 cells (Supplementary Fig. S3B) but increased doxorubicin-mediated growth inhibition in one of these three cell lines (MDA-MB-157). These data may indicate that mTORC2 inhibition may improve chemotherapy-mediated growth inhibition in *RICTOR*-amplified TNBCs.

### Rictor knockdown blocks mTORC2 signaling and cell survival without PI3K reactivation

**Figure 3:**
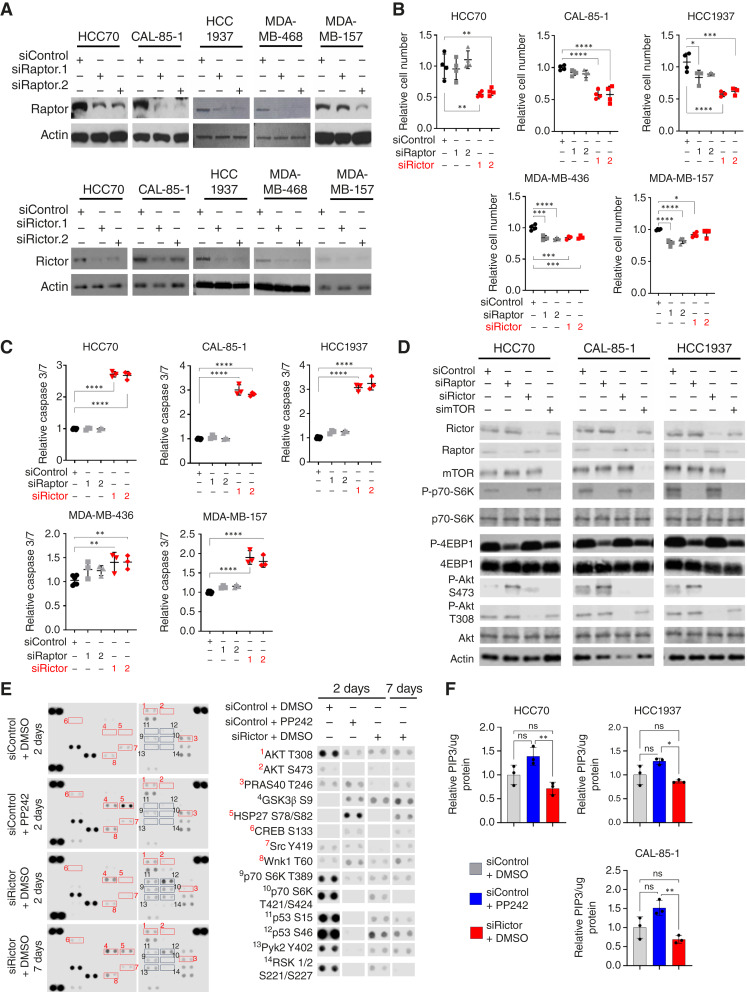
Rictor knockdown blocks mTORC2 signaling without reactivation of PI3K. **A,** Western blot analysis of cells transfected with siRaptor, siRictor, or siControl. Antibodies used are shown to the left of each panel. **B** and **C,** CellTiter-Glo was used to measure cell number (**B**) and Caspase-3/7-Glo was used to measure caspase 3/7 (cell death signaling; **C**) at 96 hours following siRNA transfection. Each point represents the average value of 3 replicates and midlines are the average values ± SD, normalized to the average value of siControl-transfected samples. One-way ANOVA was used. **D,** Western blot analysis of cells collected 72 hours after transfection. Antibodies used are shown at the left of each panel. All siRNA sequences designed against *RAPTOR* and *RICTOR* were validated for their ability to knockdown target genes. **E,** Phospho-kinase protein array was used on HCC70 cells treated with PP242 (500 nmol/L for 48 hours) or after siRNA transfection (50 nmol/L for 48 hours and 7 days after transfection). **F,** PI(3,4,5)P3 ELISA was used to measure PI3K activity from cells treated with PP242 or transfected with siRNA. PI(3,5)P2 was used as a substrate. Values shown are average ± SD, normalized to the average values in DMSO-treated samples. *N* = 3. One-way ANOVA was used.

To dissect the distinct effects of mTORC1 versus mTORC2 blockade, we designed siRNAs for potent targeting of the mTORC1 cofactor *RAPTOR* (siRaptor) and the mTORC2 cofactor *RICTOR* (siRictor), confirming knockdown of targeted transcripts (Supplementary Fig. S4) and proteins (Fig. 3A; Supplementary Fig. S4C). In *RICTOR*-amplified PI3K-active HCC70, CAL-85-1, and HCC1937 cells, transfection of siRictor significantly diminished cell growth by >40% (Fig. 3B) and increased caspase 3/7 activity approximately threefold (Fig. 3C) as compared with cells transfected with nontargeting control siRNA sequences (siControl). Growth of *RICTOR*-diploid MDA-MB-436 and MDA-MB-157 cells was also significantly decreased by transfection with siRictor, although not to the robust degree seen in *RICTOR*-amplified cell lines. Caspase 3/7 activity was elevated <twofold in both cell lines. Conversely, transfection of siRaptor sequences did not affect cell number or caspase 3/7 activity in *RICTOR*-amplified HCC70 and CAL-85-1 cells and conferred a small decrease in cell number in HCC1937 cells. siRaptor transfection significantly diminished cell number by approximately 20% in *RICTOR*-diploid MDA-MB-436 and MDA-MB-157 cells. Importantly, Rictor knockdown increased paclitaxel- and doxorubicin-mediated growth inhibition and caspase 3/7 activation in a synergistic manner (Supplementary Fig. S5), consistent with the hypothesis that mTORC2 inhibition may amplify chemotherapy-induced tumor cell killing in TNBC cells.

*RICTOR*-amplified cells transfected with siRictor displayed diminished P-Akt S473 levels without affecting mTORC1 signaling (P-p70-S6K and P-4EBP1; Fig. 3D). Transfection of cells with siRaptor decreased P-p70-S6K and P-4EBP1, confirming mTORC1 inhibition upon Raptor knockdown and revealing increased PI3K-to-mTORC2 signaling as shown by elevated P-Akt S473. Similarly, siRNA-mediated mTOR knockdown (Supplementary Fig. S6) diminished both mTORC1 and mTORC2 signaling, as shown by decreased P-p70-S6K and P-Akt S473. However, incomplete inhibition of PI3K signaling is shown by persistent P-Akt T308. This suggests that mTORC1 inhibition causes resurgent PI3K signaling but that mTORC2-selective inhibition might maintain mTORC2-mediated suppression of the PI3K pathway.

To test this hypothesis, we treated *RICTOR*-amplified HCC70 cells with either siRictor or PP242 and assessed cell signaling using an antibody array (Fig. 3E; Supplementary Fig. S7). After 48 hours, PP242 robustly decreased P-p70-S6K and P-Akt S473, confirming inhibition of mTORC1 and mTORC2. However, increased phosphorylation of the Akt substrate PRAS40 (P-PRAS40 T246) in PP242-treated cells is consistent with presence of active PI3K signaling and partial Akt reactivation. In contrast, HCC70 cells transfected with siRictor displayed inhibition of mTORC2 signaling (P-Akt S473), PI3K signaling (P-Akt T308), and Akt activity (P-PRAS40 T246). These effects were evident within 48 hours of transfection and sustained through 7 days after transfection. As a more direct measure of PI3K activity, we used an *in vitro* kinase assay, measuring PI3K-mediated phosphorylation of phosphatidylinositol (3,5)-phosphate 2 to generate PI(3,4,5)P3 (PIP3) in PP242-treated *RICTOR*-amplified cells. This study revealed significantly increased PI3K activity at 48 hours after treatment with PP242 compared with treatment with siRictor (Fig. 3F). These data confirm that mTORC2 inhibition spares mTORC1 signaling and avoids resurgent PI3K signaling in TNBC cells.

### Intravenous si-NP administration achieves siRNA delivery to TNBC tumors

**Figure 4:**
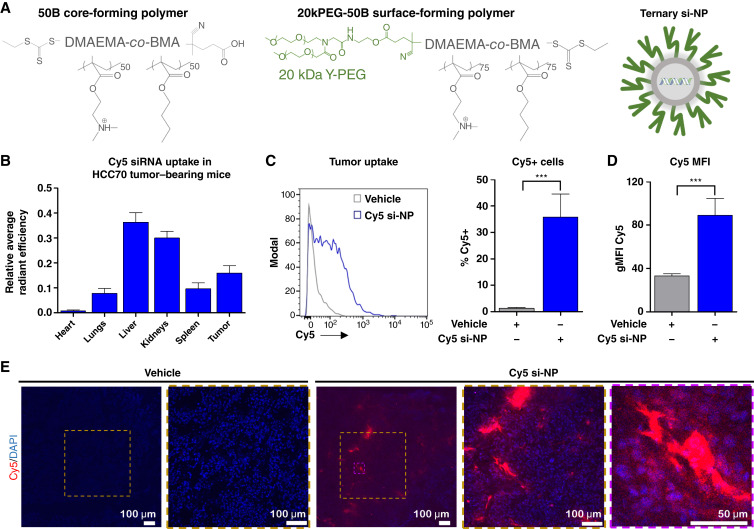
si-NP carrier technology enables siRNA delivery to TNBC tumors. **A,** Schematic representation of core-forming 50B polymer and surface-forming 20kPEG-50B polymer, and their assembly with siRNA to generate the si-NP formulation. **B,** Cy5 fluorescence was measured *ex vivo* within organs and tumors collected from HCC70 tumor–bearing mice 24 hours after delivery of Cy5-si-NPs (1 mg/kg siRNA, i.v.). Values represent the average ± SD, *N* = 6. **C,** Flow cytometric quantitation of Cy5+ cells, with representative histogram shown on left and % Cy5+ on right. **D,** Cy5 geometric mean fluorescence intensity (gMFI). Data were measured from cells dissociated from HCC70 tumors 24 hours after delivery of Cy5-si-NP (1 mg/kg, i.v.) or vehicle. Values represent the average ± SD, *N* = 5–6. An unpaired *t* test was used. **E,** Confocal microscopy to visualize Cy5-siRNA in HCC70 tumor cryosections collected 24 hours after delivery of Cy5-siNP. Nuclei stained with DAPI.

To test the efficacy of *in vivo RICTOR* silencing and selective mTORC2 inhibition in TNBC tumors, we utilized previously optimized si-NPs (ref. 32). These si-NPs are comprised of three components: the siRNA cargo and two polymers, one that forms the NP core and one that forms the NP surface. Both polymers contain a cationic and endosomolytic polymer block made up of a random copolymer of equal molar quantities of DMAEMA and BMA (50B). The NP core-forming polymer is a single block made up of purely 50B, the surface-forming polymer is an amphiphilic diblock polymer containing a 20 kDa PEG block that provides stealth shielding to the siRNA cargo (20kPEG-50B), a feature that improves siRNA delivery to tumors *in vivo* (Fig. 4A; ref. 39).

We confirmed that HCC70 tumor cells take up si-NPs carrying fluorescent tetramethylrhodamine-conjugated siRNA using flow cytometry (Supplementary Fig. S8). Biodistribution and tumor delivery of si-NPs were evaluated in HCC70 tumor-bearing mice using si-NPs formulated with Cy5-tagged siRNA and delivered at 1 mg/kg intravenously. After 24 hours, Cy5 fluorescence was measured in organs and tumors, revealing that 16% of total Cy5 fluorescence was localized within tumors (Fig. 4B). Cy5 siRNA was also detected in the liver (36%) and kidney (30%). This represents a significant improvement over previous ternary si-NP formulations that accumulate primarily in kidneys (40), in which si-NPs are destabilized, allowing excretion of released, free siRNA through urine. Flow cytometry of disaggregated tumors was done to detect Cy5-siRNA uptake in >35% of tumor cells (Fig. 4C) and a >2.5-fold increase in Cy5 geometric mean fluorescent intensity (Fig. 4D) compared with vehicle-treated tumors. Fluorescence microscopy of frozen tumor sections confirmed Cy5-siRNA accumulation in tumors from mice treated with si-NPs (Fig. 4E). Notable pockets of high siRNA accumulation were seen in the tumor periphery, which may provide a depot for siRNA tumor delivery over an extended period of time.

### Selective mTORC2 inhibition using siRictor-NPs increases TNBC tumor cell death

We loaded si-NPs with *RICTOR*-targeting siRNA (siRictor-NPs). We previously used CRISPR-mediated gene editing in MDA-MB-231 cells to tag endogenous Rictor with a HiBiT peptide (Rictor^HiBiT^; ref. 32) enabling high-throughput Rictor protein quantitation using split nano-luciferase complementation. MDA-MB-231-Rictor^HiBiT^ cells treated with siRictor-NPs exhibited up to 60% diminished Rictor^HiBiT^ levels within 24 hours, which were maintained over multiple days and functionally increased cell apoptosis at 7 days after treatment (Supplementary Fig. S9), supporting further study of siRictor-NPs.

**Figure 5:**
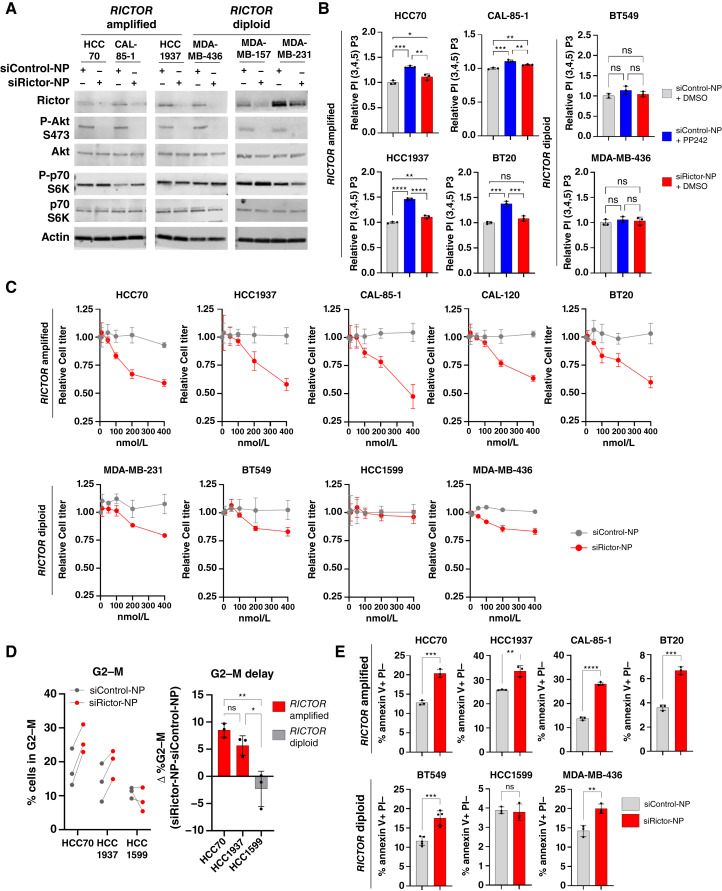
On target silencing and therapeutic implications of TNBC treatment with custom siRictor-NPs. **A,** Western blot analysis of cells treated with si-NPs (100 nmol/L). Antibodies used are shown on the left of each panel. **B,** PI(3,4,5)P3 ELISA following *in vitro* PI3K assay from cells treated with si-NPs (100 nmol/L) or PP242 (500 nmol/L). Each point is the average of 2–3 technical replicates. Midline is average ± SD normalized to the average value of DMSO-treated samples. One-way ANOVA was used. **C,** CellTiter-Glo assay to measure cell viability in cells treated with a dose curve of si-NP. Each point is the average of 3 biological replicates. Midline is average ± SD normalized to the average value of siControl-NP–treated samples. **D,** Cells were treated with 200 nmol/L si-NPs, and the proportion of cells in the G_2_–M cell-cycle phase was assessed by PI staining. Midline is average of 3 biological replicates normalized to the average value of siControl-NP–treated samples. One-way ANOVA was used. **E,** Annexin V staining to measure cell apoptosis in *RICTOR*-amplified and *RICTOR*-diploid cells treated with 200 nmol/L si-NPs. Midline is the average of 3 biological replicates. An unpaired *t* test was used.

*RICTOR*-amplified (HCC70, CAL-85-1, and HCC1937) and *RICTOR*-diploid (MDA-MB-436, MDA-MB-157, and MDA-MB-231) cells were treated with siRictor-NPs or siControl-NPs in serum-free media for 24 hours (Fig. 5A). mRNA level knockdown of *RICTOR* using siRictor-NPs was confirmed in HCC70 cells (Supplementary Fig. S10). Western blot analysis of cells collected at 96 hours after treatment revealed decreased Rictor expression and decreased P-Akt S473, consistent with mTORC2 inhibition in each cell line tested (Fig. 5A). *In vitro* PI3K activity was measured in lysates collected from cells treated with siControl-NP and siRictor-NP and was compared with cells treated with the dual mTORC1/2 kinase inhibitor PP242. Although PP242-treated cells significantly increased PI3K activity, cells treated with siRictor-NP did not (Fig. 5B), confirming that selective mTORC2 inhibition by siRictor-NPs avoids resurgent PI3K signaling in *RICTOR*-amplified TNBC cells. Interestingly, neither PP242 nor siRictor-NP treatment increased PI3K signaling in *RICTOR*-diploid cell lines, BT549 and MDA-MB-436.

We found that siRictor-NP treatment diminished viability of *RICTOR*-amplified TNBC cells in a dose-dependent manner following treatment (Fig. 5C). Cell growth was also diminished in most *RICTOR*-diploid cell lines tested (MDA-MB-231, BT549, and MDA-MB-436), although not to the extent seen in *RICTOR*-amplified cell lines (< 25% growth inhibition at 400 nmol/L siRictor-NP). These modest levels of growth inhibition, particularly in the MDA-MB-231 and BT549 cells, may be due to the high levels of the Rictor protein as well as additional activating PI3K alterations present in these lines (41). Sensitivity to siRictor treatment in the MDA-MB-231 cells is furthermore consistent with previous reports (42–45). It is important to note that MDA-MB-436 cells also displayed dose-dependent inhibition of cell growth from siRictor-NP treatment, despite being a *RICTOR*-diploid cell line that has no obvious PI3K/mTOR pathway alterations. These results suggest that Rictor inhibition may also have a therapeutic role in the setting of *RICTOR*-diploid TNBC.

A growing body of evidence suggests that mTORC2 signaling may promote cell-cycle progression during S, G_2_, or early mitosis. We used BrdU incorporation assays to more directly assess DNA synthesis following siRictor-NP treatment and found that siRictor-NPs significantly decreased BrdU-positive cells in *RICTOR*-amplified, but not *RICTOR*-diploid, cells (Supplementary Fig. S11). We measured the impact of siRictor-NP on the cell cycle in *RICTOR-*amplified HCC70 and HCC1937 cells, finding that siRictor-NP treatment increased the proportion of *RICTOR*-amplified cells in the G_2_–M phase but did not do the same in *RICTOR*-diploid HCC1599 cells (Fig. 5D; Supplementary Fig. S12). Significant differences in the S-phase were not seen in siRictor-NP–treated cells. We also quantified siRictor-NP effect on inducing apoptotic cells using annexin V staining, finding cell death to be significantly increased in *RICTOR*-amplified cell lines after siRictor-NP treatment (Fig. 5E). siRictor-NP treatment also increased cell apoptosis in *RICTOR*-diploid BT549 and MDA-MB-436 cells but not in HCC1599 cells. These findings suggest that mTORC2-selective targeting may decrease tumor growth through effects both on cell proliferation and cell death. Together, these data confirm that the delivery of our custom siRictor-NPs achieves mTORC2 signaling inhibition and confers long-lasting growth inhibition and cell death in TNBC cell lines.

**Figure 6:**
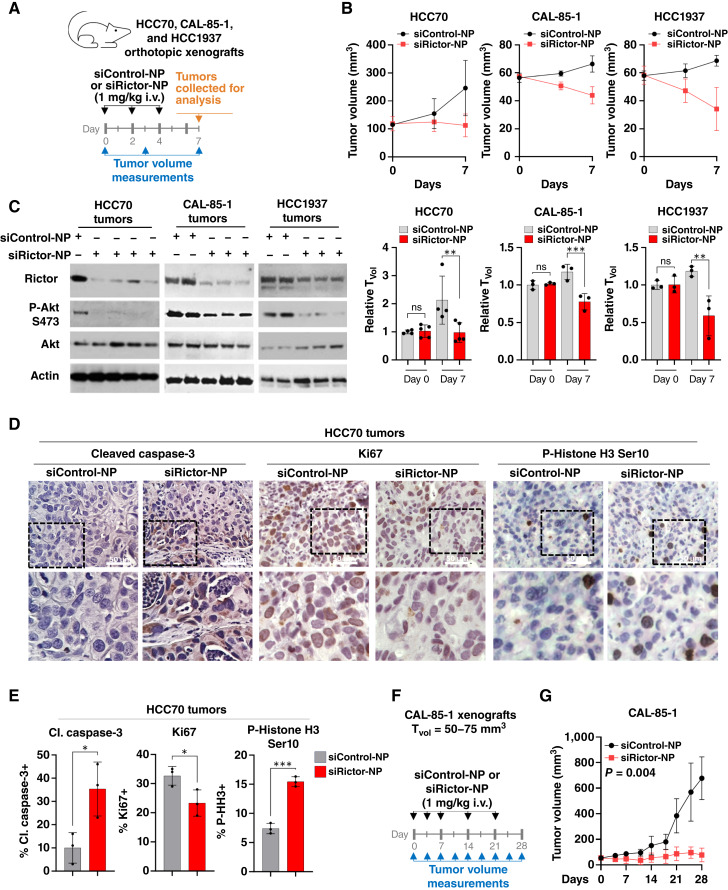
*In vivo* delivery of siRictor-NPs blocks mTORC2 in the tumors and increases tumor cell death. **A,** HCC70 (*N* = 4–5), CAL-85-1 (*N* = 3), and HCC1937 (*N* = 3) tumor–bearing mice were treated with si-NPs intravenously, and tumors were harvested for molecular analysis on day 7. Schematic timeline of treatment schedule is shown. **B,** Tumor volumes (T_vol_) were tracked to the study endpoint. **C,** Western blot analysis of whole-tumor lysates. Antibodies used are shown to the left of each panel. **D** and **E,** Quantitation of the percentage of tumor cells that are Ki67+, cleaved (Cl.) caspase-3+, and P-Histone H3+ (P-HH3) based on IHC of HCC70 tumor sections. For quantitation, each point represents the average of 4–5 randomly chosen fields from a single tumor. Midline is the average ± SD of *N* = 3 tumors. An unpaired *t* test was used. **F** and **G,** CAL-85-1 tumor–bearing mice (*N* = 4) were treated with si-NPs intravenously, and tumor volume was measured. Schematic timeline (**F**) of treatment schedule and tumor growth (**G**) is shown. Significance calculated using an unpaired *t* test of AUC analysis.

We next assessed the acute impact of selective mTORC2 inhibition in TNBC tumors using siRictor-NPs *in vivo*. Athymic mice harboring orthotopic HCC70, CAL-85-1, and HCC1937 xenografts were randomized into treatment groups to receive siRictor-NP (1 mg/kg, i.v.) or siControl-NPs when tumor volume reached 100 mm^3^ (HCC70) or 50 mm^3^ (CAL-85-1 and HCC1937; Fig. 6A; Supplementary Fig. S13). Mice were treated on days 0, 2, and 4, and tumors were analyzed on day 7 for Rictor silencing. Tumor volume measurements revealed decreased growth in tumors treated with siRictor-NPs in all three *RICTOR*-amplified models (Fig. 6B). Western blot analysis of tumor lysates confirmed Rictor knockdown and decreased P-Akt S473 (Fig. 6C), confirming mTORC2 inhibition in tumors from mice treated with siRictor-NPs. IHC staining of HCC70 tumors for Ki67 revealed a modest but significant decrease in cell proliferation in siRictor-NP–treated tumors (Fig. 6D and E). Cleaved caspase-3 IHC staining was increased >fourfold over siControl-NP–treated tumors, demonstrating increased apoptosis in siRictor-NP–treated tumors. This result correlated with an increase in acellular area quantified in hematoxylin and eosin–stained histologic sections of tumors from siRictor-NP–treated mice compared with tumors from siControl-NP–treated mice (Supplementary Fig. S13A). siRictor-NP treatment increased tumor P-Histone H3 Ser10 (Fig. 6D and E), consistent with the idea that Rictor silencing may cause mitotic delay. These early molecular endpoints suggested that *in vivo* siRictor-NPs treatment reduced tumor growth and tumor cell survival, motivating further testing of siRictor-NPs in longer-term treatment responses. CAL-85-1 tumor–bearing mice were treated with siRictor-NPs intravenously over the course of 4 weeks, revealing robust tumor growth inhibition compared with siControl-NP–treated tumors (Fig. 6F and G).

Mouse plasma of HCC70 tumor–bearing mice collected on treatment day 28 was assessed for markers of kidney (blood urea nitrogen) and liver (alanine aminotransferase and aspartate aminotransferase) damage. However, these markers remained at basal levels in mice treated with siControl-NPs or siRictor-NPs (Supplementary Fig. S13B). Serum glucose levels were also similar in siRictor-NP–treated mice. It should be noted that siRNA sequences used herein are directed at human Rictor mRNA rather than mouse Rictor mRNA, although we have previously shown that the delivery of our si-NPs harboring mouse Rictor siRNA does not result in toxicities (32). These collective data confirm that the si-NP formulation achieves delivery of active Rictor siRNA to the tumor for selective, therapeutic mTORC2 inhibition without causing systemic toxicities.

### Therapeutic delivery of siRictor-NPs produces robust TNBC growth inhibition and potentiates paclitaxel-mediated tumor cell killing *in vivo*

**Figure 7:**
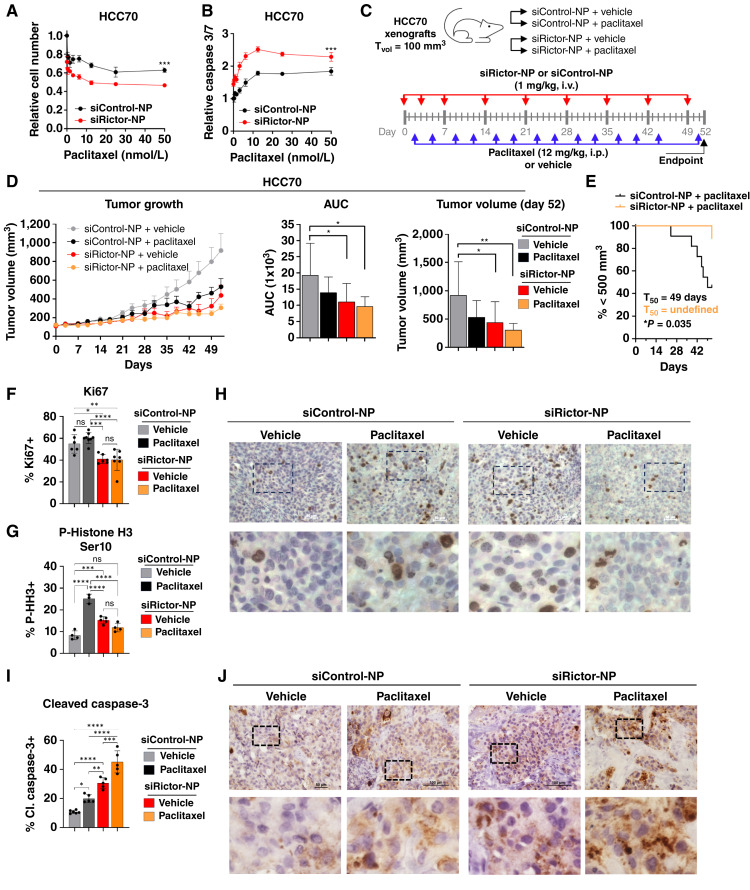
siRictor-NPs treatment synergizes with chemotherapy to diminish tumor cell survival and tumor growth in TNBC. **A** and **B,** HCC70 cells were treated with 100 nmol/L si-NPs ± paclitaxel in increasing doses. Cell viability was measured by CellTiter-Glo 2 days after chemotherapy addition (**A**), and caspase 3/7 activity was measured by Caspase-Glo 1 day after chemotherapy addition (**B**). Statistical significance was calculated based on AUC, in which midline is the average ± SD and *N* = 3. An unpaired *t* test was used. **C–I,** HCC70 tumor–bearing mice (*N* = 9–11) were treated with si-NPs intravenously and paclitaxel intraperitoneally, and the tumors were monitored until harvest for molecular analysis on day 52. **C,** Schematic timeline of treatment schedule. **D,** Tumor volume was measured throughout the 52-day treatment period. Values shown on tumor growth curve (left) are average ± SE. Statistical significance was calculated based on AUC (right), in which midline is the average ± SD and *N* = 9–11 tumors. Endpoint tumor volume was calculated at day 52. Midline is the average ± SD. One-way ANOVA was performed with the Tukey multiple comparison *post hoc* test. **E,** Kaplan–Meier analysis of tumor-bearing mice, defining survival as tumor volume under 500 mm^3^ (paclitaxel-treated mice). Average time to exceed the defined tumor volume (T_50_) for each group is shown within panel. Significance calculated using the log-rank (Mantel–Cox) test. **F,** Quantitation of the percentage of tumor cells that are Ki67+ (*N* = 7–10 tumors) based on IHC of tumor sections. For quantitation, each point represents an average of 4–5 randomly chosen fields from a single tumor. Midline is the average ± SD. One-way ANOVA was performed with the Tukey multiple comparisons *post hoc* test. **G** and **H**, Quantitation of the percentage of tumor cells that are P-Histone H3 (P-HH3) Ser10+ (*N* = 3–4) (**G**) based on IHC of tumor sections (**H**). For quantitation, each point represents an average of 4–5 randomly chosen fields from a single tumor. Midline is the average ± SD. One-way ANOVA was performed with the Tukey multiple comparisons *post hoc* test. **I** and **J,** Quantitation of the percentage of tumor cells that are cleaved (Cl.) caspase-3+ (**I**) based on IHC of tumor sections (**J**). For quantitation, each point represents an average of 4–5 randomly chosen fields from a single tumor. Midline is the average ± SD of *N* = 5–6 tumors. One-way ANOVA was performed with the Tukey multiple comparison *post hoc* test.

HCC70 cells treated with an increasing dose of doxorubicin or paclitaxel showed progressively decreased cell viability and increased apoptosis, which was increased further upon combined treatment with siRictor-NPs (Fig. 7A and B; Supplementary Fig. S14). These data motivated us to probe the *in vivo* therapeutic impact of siRictor-NPs combined with chemotherapy. We randomized HCC70 tumor-bearing mice into treatment groups for siControl-NP or siRictor-NP (1 mg/kg, i.v.). Beginning when tumors reached a volume of 100 mm^3^, mice were treated in the first week on days 0, 3, and 7 (Fig. 7C). Thereafter, mice were treated once weekly through 7 weeks (day 49), measuring tumors through day 52. We randomly allocated mice into treatment groups to receive vehicle or the chemotherapeutic agent paclitaxel (12 mg/kg, i.p., twice weekly for 6 weeks and then once weekly starting at day 44), testing the hypothesis that selective mTORC2 blockade in TNBC tumors may potentiate chemotherapy-mediated tumor cell killing. We found that siRictor-NP treatment resulted in HCC70 tumor growth inhibition, decreasing final tumor volume to approximately 57% of tumors from mice treated with siControl-NP (Fig. 7D; Supplementary Fig. S15). Paclitaxel-treated tumors were growth inhibited compared with vehicle-treated tumors, although not to the level of growth inhibition seen with siRictor-NPs. Importantly, the combination of siRictor-NPs with paclitaxel blocked tumor growth to the greatest extent, with final tumor volume decreasing to 33% of control tumors. Notably, 6 of 11 paclitaxel-treated tumors exceeded 500 mm^3^ volume before day 52, with an average latency of 49 days. However, the combination treatment of siRictor-NP + paclitaxel decreased this incidence (one of nine tumors) by extending the latency period and delaying tumor progression (Fig. 7E). Histologic analyses at the study endpoint confirmed that the decreased tumor growth seen in siRictor-NP–treated tumors resulted from a greater induction of tumor cell death (Fig. 7F–J); Supplementary Fig. S15D and S15E). As expected, paclitaxel treatment maintained Ki67 expression at the level seen in control tumors because of mechanistic growth arrest in mitosis. However, tumor cell proliferation was decreased in siRictor-NP tumors and similarly diminished in combination-treated tumors (Fig. 7F). Histologic analysis confirmed these results, revealing a >twofold increase in P-Histone H3 Ser10 in paclitaxel-treated tumors (Fig. 7G and H). This marker is indicative of cells stalled in mitosis and was diminished significantly upon combination with siRictor-NP (Fig. 7G and H). The mechanism underlying this observation remains unclear. Importantly, siRictor-NP treatment induced an approximate threefold increase in tumor cell apoptosis compared with control-treated tumors, which was further increased upon combination with paclitaxel (Fig. 7I and J) Mouse body weight was monitored to confirm that si-NP and paclitaxel treatment was well tolerated throughout the study, with no notable loss in mass (Supplementary Fig. S15F). Collectively, these data show that intravenous delivery of siRictor-NPs blocks mTORC2 activity to therapeutic levels and synergizes with paclitaxel chemotherapy to enhance tumor cell killing.

## Discussion

PI3K/mTOR signaling pathways are frequently altered in TNBC, contributing to chemoresistance. Here, we achieve selective mTORC2 blockade in PI3K-active TNBCs using a novel nanomedicine for tumor delivery of Rictor-targeting siRNAs. We show that selective mTORC2 inhibition decreases TNBC cell survival and diminishes tumor growth *in vivo*. Importantly, we show that mTORC2 increases TNBC sensitivity to chemotherapy, improving tumor growth inhibition in *in vivo RICTOR*-amplified TNBC models.

### PI3K/mTOR/AKT pathway inhibitors in TNBC and a role for selective mTORC2 inhibition

Patients with premetastatic TNBC continue to lack molecularly targeted therapeutic options, making NAC and tumor resection surgery the clinical standard of care. Although TNBCs may be more sensitive to chemotherapies than other breast cancer subtypes, as few as 30% of patients with TNBC may experience a pathologic complete response following NAC (2, 46). Aberrant PI3K pathway alterations occur frequently in TNBCs, often as activating *PIK3CA* mutations or *PTEN* loss-of-function alterations(47). Interestingly, our CCLE analysis revealed that PI3K pathway alterations (PIK3CA mutations and/or PTEN loss) co-occurred in 90% of TNBC cases with *RICTOR* amplification. This further suggests that PI3K pathway alterations are a frequent molecular event in TNBC and that the respective contributions of *RICTOR*, *PIK3CA*, and *PTEN* to mTOR hyperactivation may drive tumor progression. Although data shown here are based on genomic analysis of immortalized cell lines and therefore may not reflect clinical TNBC populations, *RICTOR* amplification has been shown to co-occur with PI3K pathway alterations in TNBC and other cancers at a relatively high frequency (48, 49). Thus, there is intense clinical interest in PI3K pathway inhibition. Pan-PI3K inhibitors have achieved limited clinical success because of associated toxicities (50), but strategies to inhibit downstream effectors of this pathway, such as the mTOR complexes, have the potential to be more selective. Data shown here demonstrate that selective mTORC2 inhibition is a potential vulnerability within the pathway, particularly when sparing mTORC1 signaling.

Everolimus is an allosteric inhibitor selective for mTORC1 activity blockade that is approved for the treatment of other breast cancer subtypes. However, favorable outcomes were not seen for patients with TNBC treated with everolimus, even when combined with chemotherapy (14, 15, 51). mTORC1 signaling is capable of IRS1-mediated negative feedback regulation of PI3K activity, meaning that mTORC1 inhibitors might actually increase PI3K signaling. The relief of IRS1 mediated by mTORC1 inhibition may explain the relative failure of this strategy in the TNBC realm. Dual mTORC1/2 inhibitors can block the impact of resurgent PI3K activation on mTORC2 but still allow activation of other PI3K effectors, potentially limiting their therapeutic efficacy. Although many dual mTORC1/2 inhibitors are still in clinical development and may show promise, the mTOR kinase inhibitor AZD2014 combined with hormone therapy failed to improve progression-free survival or confer an advantage over everolimus in a phase II trial (52). Here, we utilized siRNA technology to selectively inhibit mTORC2 activity through mRNA-level Rictor ablation, a strategy that avoids resurgent PI3K activity (Figs. 3F and 5D) and enables precise assessment of mTORC2 targeting in TNBC. Encapsulation of our siRNA in a translationally-relevant si-NP nanomedicine furthermore allowed therapeutic testing of selective and potent mTORC2 inhibition in an *in vivo* TNBC setting.

### siRictor as a tool for assessing the signaling impact of selective mTORC2 inhibition in *RICTOR*-amplified TNBC

The lack of mTORC2-selective inhibitors stymies attempts to understand the impact of mTORC2 inhibition, including feedback through compensatory pathways. However, we leverage Rictor RNAi technology as a powerful tool to gain these insights. The role of Rictor/mTORC2 for Akt S473 phosporylation to promote cancer survival is well established, but mTORC2 controls many other effectors such as AGC kinase SGK and PKC, which can propagate effects to oncogenic signaling loops (24, 53, 54). Our studies probe kinase activity more broadly in *RICTOR*-amplified TNBC, which may benefit greatly from selective mTORC2 inhibition, through determination of substrate phosphorylation following siRictor treatment (Fig. 3E). For example, siRictor treatment creates prolonged inhibition of AKT S473 phosphorylation as well as phosphorylation of its downstream effector, PRAS40, at T246. As Akt-mediated phosphorylation of PRAS40 at T246 permits mTORC1 activation (55, 56), siRictor treatment effectively avoids resurgent mTORC1 activity in this manner. Another Akt target, GSK3α, was unaffected by siRictor treatment, but we observed an increase in GSK3β S9 phosphorylation (Supplementary Fig. S7). Akt-S473 phosphorylation is not required for GSK3β S9 phosphorylation (57), so modulation of this substrate may suggest perturbations in other signaling pathways following siRictor treatment. These and other signaling events can help inform combination therapies that will cooperate with mTORC2 inhibition to combat a heterogeneous disease such as TNBC. This is of particular importance when attempting to use *RICTOR* levels as potential stratification criteria for senstivity to siRictor therapy, as our analyses in *RICTOR*-diploid cell lines revealed that mTORC2 inhibition conferred small but appreciable efficacy in some cell lines expressing low *RICTOR*. Additional studies are needed to rigorously assess the signaling mechanisms affected by selective mTORC2 inhibition.

### mTORC2 inhibition as a promising therapeutic strategy to enhance chemotherapy effects

An increasing number of studies suggest that targeting the PI3K/mTOR/Akt pathway may sensitize cancer cells to chemotherapy and that mTORC2 may play an important role in enabling this effect (58, 59). In leukemia cells, PP242 treatment, but not rapamycin treatment, sensitized cells to DNA damage induced by cisplatin, a DNA crosslinker chemotherapeutic. This effect was associated with an mTOR-specific regulation of FANCD2, a member of the Fanconi anemia DNA repair pathway, which was downregulated upon PP242 treatment but not rapamycin treatment (60). This may suggest that mTORC2 controls end-joining DNA repair through FANCD2 expression. Furthermore, mTORC2 blockade combined with paclitaxel chemotherapy may produce cooperative effects that are rooted in the mechanisms of action that are shared between these two therapies. Emerging data suggest that mTORC2 may regulate microtubule organization by way of its more well-known roles in actin remodeling through Rho and Rac effector pathways (53, 61). Studies on the mTORC2 effect on endothelial cell elongation revealed that dual mTORC1/2 inhibition, but not selective mTORC1 inhibition, blocked elongation by way of affecting microtubule organization (62). Importantly, this effect was recapitulated by microtubule stabilization induced by paclitaxel treatment, suggesting that mTORC2 inhibition may play a similar role in spindle microtubule dynamics. mTORC2 may also affect mitotic progression through its downstream effector NDRG1, a known regulator of centrosome activity and chromosome segregation during mitosis (63). However, the molecular impact of mTORC2 on NDRG1-mediated chromosome segregation is not yet explored, and further studies are warranted to elucidate the underlying mechanisms of mTORC2 activity that may drive chemotherapy sensitization by affecting mitotic progression. Although we found an accumulation of tumor cells in mitosis upon treatment with siRictor-NP (Fig. 5D), the mechanism underlying this observation remains unclear, as is the wider role of mTORC2 in mitotic progression. It is furthermore likely that the effect of mTORC2 on chemotherapy sensitization is in fact multi-factorial, given the key role of mTORC2 signaling in activation of pro-survival pathways such as Akt. In this work, we show that siRictor-NP–mediated mTORC2 blockade in the tumor strongly inhibits TNBC tumor growth. siRictor-NPs have an even stronger effect when combined with paclitaxel and block tumor growth to a greater extent than what is achieved by paclitaxel alone. Our data therefore point to mTORC2 inhibition as a promising combinatorial strategy for TNBC treatment.

### Conclusion

In this work, we provided evidence that therapeutic targeting of Rictor to enable selective mTORC2 inhibition can be an efficacious strategy in TNBC. We utilized clinically relevant mTOR inhibitors capable of mTORC1-selective or dual mTORC1/2 blockade to elucidate the important role of mTORC2 signaling in tumor cell survival and dissected their effects in various TNBC cells harboring *RICTOR* amplification. Our work showed that *RICTOR*-amplified TNBCs are particularly senstive to mTORC2 blockade, making this a viable, molecularly targeted therapy for certain subtypes of TNBC, but could not rule out a potential yet modest benefit for mTORC2 targeting in *RICTOR*-diploid cells. We further validated RNAi-mediated silencing of Rictor as a selective inhibitor of mTORC2 activity that provides therapeutic benefit while not perturbing mTORC1 signaling or encouraging PI3K activity. Finally, we applied a translational nanotechnology that enables effective delivery of our Rictor RNAi to TNBC tumors *in vivo* to achieve robust mTORC2 inhibition. Using this Rictor nanomedicine, we show that established *RICTOR*-amplified PI3K-active TNBC tumors benefit from selective mTORC2 inhibition and that this strategy cooperates with chemotherapy.

## Supplementary Material

Supplemental Table 1 RICTOR-amplified and RICTOR-diploid TNBC cell lines

Supplemental Table 2 siRNA sequences

Supplemental Table 3 Primer sequences

Supplemental Schematic 1 PI3K-mTOR signaling pathway

Supplemental Figure S1 Dataset analyses

Supplemental Figure S2 RAD001 and PP242 treatment effects on mTORC1 and mTORC2 effectors

Supplemental Figure S3 Chemotherapy response of TNBC cell lines

Supplemental Figure S4 Validation of target silencing activity of siRictor and siRaptor

Supplemental Figure S5 Combination treatment of siRictor and chemotherapies

Supplemental Figure S6 Development and cell growth testing of siMTOR

Supplemental Figure S7 Phospho-kinase array

Supplemental Figure S8 Tumor cell uptake of si-NPs

Supplemental Figure S9 siRictor-NPs on target silencing and induction of apoptosis in TNBCs

Supplemental Figure S10 siRictor-NP mediated mRNA knockdown

Supplemental Figure S11 siRictor-NP treatment impact on cell proliferation

Supplemental Figure S12 siRictor-NP treatment impact on cell growth

Supplemental Figure S13 Extended tumor data for short-term siRictor-NP therapeutic effects in HCC70 tumor-bearing mice

Supplemental Figure S14 siRictor-NPs combination with chemotherapy

Supplemental Figure S15 Extended tumor data for siRictor-NP combination with paclitaxel in HCC70 tumor-bearing mice
