# Supplementary material for: Induction of Triple-Negative Breast Cancer Cell Death and Chemosensitivity Using mTORC2-Directed RNAi Nanomedicine
Source: Cancer Res Commun. 2025 Mar 19;5(3):458–76. doi: 10.1158/2767-9764.CRC-24-0261 (PMC11921867; doi:10.1158/2767-9764.CRC-24-0261)
Supplement: Supplemental Table 1 — RICTOR-amplified and RICTOR-diploid TNBC cell lines [file crc-24-0261_supplemental_table_1_suppst1.pdf]

Supplemental Table 1. *RICTOR*-amplified and *RICTOR*-diploid TNBC cell lines.

Cell line	*RICTOR* ploidy	Rictor mutation	Rictor mRNA	Rictor protein	Additional activating PI3K alteration
**HCC70**	**Amplified**	None	**high**	**high**	*PIK3CA* mut; *PTEN* mut
**CAL-85-1**	**Amplified**	None	med	med	*PIK3CA* amp/low exp; *PIK3R1* low exp; *INPP4B* high
**HCC1937**	**Amplified**	none	med	med	*PIK3CA* amp; *PTEN* loss
**BT20**	**Amplified**	none	med	med	*PIK3CA H1047R*; *PTEN* loss
**CAL-120**	**Amplified**	none	med	med	No: *PIK3CA* high exp; INPP4B high
**BT549**	Diploid	none	**high**	**high**	*PTEN* loss
**MDA-MB-231**	Diploid	none	**high**	**high**	*PIK3CA* high; *PIK3R1* high; *KRAS* amp
**MDA-MB-436**	Diploid	none	med	med	none
**HCC1599**	Diploid	none	med	med	*PIK3CA* amplification; *PIK3R1* loss
**HCC1187**	Diploid	none	med	med	No: low *PIK3CA* expression
**MDA-MB-157**	Diploid	none	n.d.	n.d.	No CNVs or point mutations
