# Supplementary material for: Induction of Triple-Negative Breast Cancer Cell Death and Chemosensitivity Using mTORC2-Directed RNAi Nanomedicine
Source: Cancer Res Commun. 2025 Mar 19;5(3):458–76. doi: 10.1158/2767-9764.CRC-24-0261 (PMC11921867; doi:10.1158/2767-9764.CRC-24-0261)
Supplement: Supplemental Table 2 — siRNA sequences [file crc-24-0261_supplemental_table_2_suppst2.pdf]

Supplemental Table 2. siRNA sequences

Oligonucleotide	Sequence (5' to 3')
**Control sense**	fU*mU*fC mU fC mC fG mA fA mC fG mU fG mU fC mA fC*mG*fU
**Control antisense**	5PHO mA*fC*mG fU mG fA mC fA mC fG mU fU mC fG mG fA mG*fA*mA
**Rictor.1 sense**	fG*mA*fA mG fA mU fU mU fA mU fU mG fA mG fU mC fC*mU*fA
**Rictor.1 antisense**	5PHO mU*fA*mG fG mA fC mU fC mA fA mU fA mA fA mU fC mU*fU*mC
**Rictor.2 sense**	GAC ACA AGC ACU UCG AUU AUU
**Rictor.2 antisense**	UAA UCG AAG UGC UUG UGU CUU
**Raptor.1 sense**	fU*mG*fG mC fU mA fG mU fC mU fG mU fU mU fC mG fA*mA*fA
**Raptor.1 antisense**	5PHO mU*fU*mU fC mG fA mA fA mC fA mG fA mC fU mA fG mC*fC*mA
**Raptor.2 sense**	fU*mG*fG mA fG mA fA mG fC mG fU mG fU mC fA mG fA*mU*fA
**Raptor.2 antisense**	5PHO mU*fA*mU fC mU fG mA fC mA fC mG fC mU fU mC fU mC*fC*mA
**mTOR sense**	fG*mG*fC mC fA mU fA mG fC mU fA mG fC mC fU mC fA*mU*fA
**mTOR antisense**	5PHO mU*fA*mU fG mA fG mG fC mU fA mG fC mU fA mU fG mG*fC*mC
**Modifications:** f = backbone 2'Fluoro; m = backbone 2'O-Methyl; * = phosphorothioate bond; 5PHO = 5' phosphorylation;<br>Cy5 fluorophore tagged on 5' end of Control sense strands	
