# Supplementary material for: Induction of Triple-Negative Breast Cancer Cell Death and Chemosensitivity Using mTORC2-Directed RNAi Nanomedicine
Source: Cancer Res Commun. 2025 Mar 19;5(3):458–76. doi: 10.1158/2767-9764.CRC-24-0261 (PMC11921867; doi:10.1158/2767-9764.CRC-24-0261)
Supplement: Supplemental Table 3 — Primer sequences [file crc-24-0261_supplemental_table_3_suppst3.pdf]

Supplemental Table 3. Primer sequences

Primer	Sequence (5' to 3')
GAPDH forward	TCT TTT GCG TCG CCA GCC
GAPDH reverse	TGA CCA GGC GCC CAA TAC
RICTOR forward	GTG CCA CAT ATG GGG GTT CA
RICTOR reverse	GTT CCA GAT GGA AGA CCT CCT G
RAPTOR forward	TCG TCA AGT CCT TCA AGC AG
RAPTOR reverse	GGG TGA TTT GGG TTG ATT GC
MTOR forward	GCT GTG AGG TCT GAG TTT AAG G
MTOR reverse	ATT GCC TTC TGC CTC TTA TGG
