# Supplementary material for: Induction of Triple-Negative Breast Cancer Cell Death and Chemosensitivity Using mTORC2-Directed RNAi Nanomedicine
Source: Cancer Res Commun. 2025 Mar 19;5(3):458–76. doi: 10.1158/2767-9764.CRC-24-0261 (PMC11921867; doi:10.1158/2767-9764.CRC-24-0261)
Supplement: Supplemental Schematic 1 — PI3K-mTOR signaling pathway [file crc-24-0261_supplemental_schematic_1_suppss1.pdf]

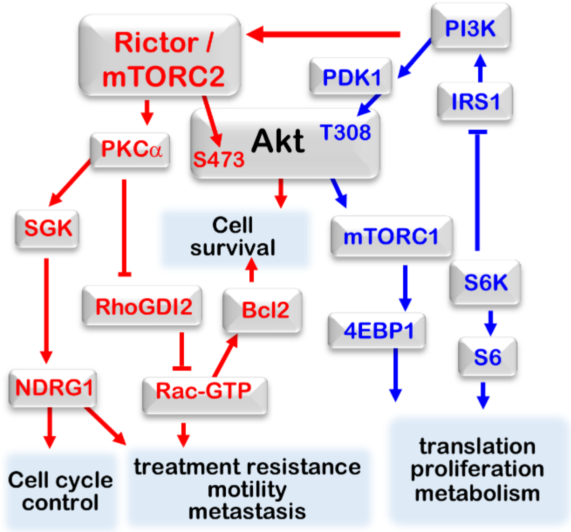

Supplemental Schematic 1. PI3K-mTOR signaling pathway.
Schematic highlights the distinct activation pathways of mTORC1 and mTORC2, their unique effectors, and mTORC1-mediated feedback inhibition of PI3K.
