# Supplementary material for: Induction of Triple-Negative Breast Cancer Cell Death and Chemosensitivity Using mTORC2-Directed RNAi Nanomedicine
Source: Cancer Res Commun. 2025 Mar 19;5(3):458–76. doi: 10.1158/2767-9764.CRC-24-0261 (PMC11921867; doi:10.1158/2767-9764.CRC-24-0261)
Supplement: Supplemental Figure S1 — Dataset analyses [file crc-24-0261_supplemental_figure_s1_suppsf1.pdf]

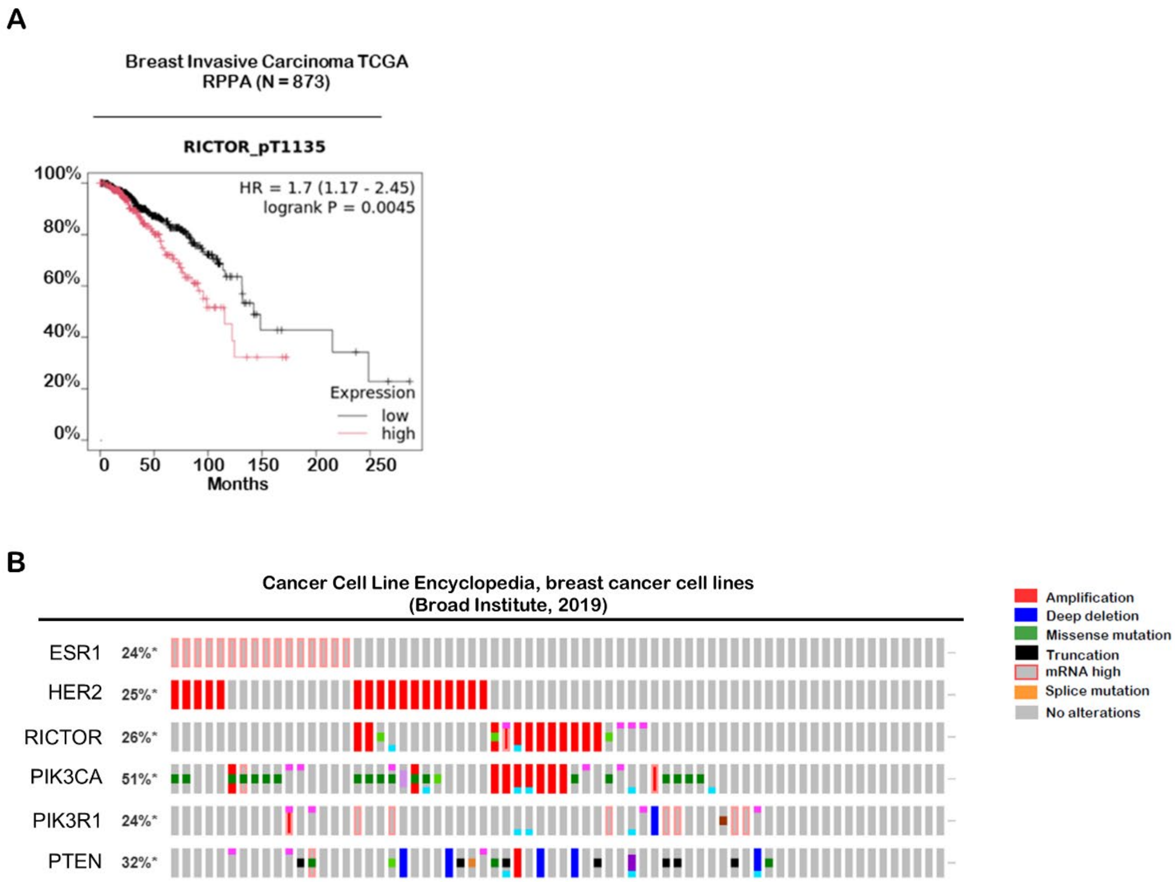

Supplemental Figure S1. Dataset analyses. A) Kaplan-Meier curves illustrating the impact of Rictor phosphorylation at T1135 (software-defined best cutoff) on OS in TCGA invasive breast cancer RPPA dataset using KmPlot software. B) TNBC cell lines were assessed for genomic copy number variations within 71 breast-derived cell lines profiled in the Cancer Cell Line Encyclopedia after excluding breast lines with ER (*ESR1*) expression and HER2 (*ERBB2*) gene amplification.
