# Supplementary material for: Induction of Triple-Negative Breast Cancer Cell Death and Chemosensitivity Using mTORC2-Directed RNAi Nanomedicine
Source: Cancer Res Commun. 2025 Mar 19;5(3):458–76. doi: 10.1158/2767-9764.CRC-24-0261 (PMC11921867; doi:10.1158/2767-9764.CRC-24-0261)
Supplement: Supplemental Figure S2 — RAD001 and PP242 treatment effects on mTORC1 and mTORC2 effectors [file crc-24-0261_supplemental_figure_s2_suppsf2.pdf]

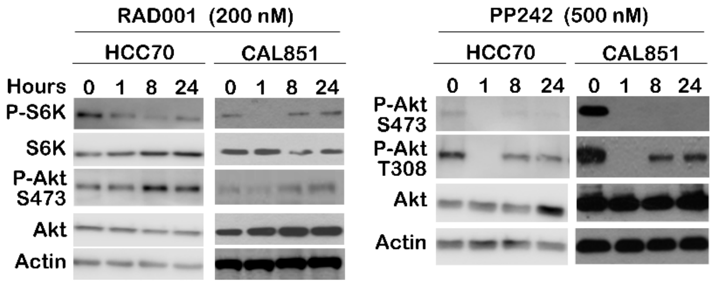

Supplemental Figure S2. RAD001 and PP242 treatment effects on mTORC1 and mTORC2 effectors. Western analysis of cells cultured in 2.5% serum that were treated with RAD001 (200 nM) or PP242 (500 nM) for 0-24 hrs.
