# Supplementary material for: Induction of Triple-Negative Breast Cancer Cell Death and Chemosensitivity Using mTORC2-Directed RNAi Nanomedicine
Source: Cancer Res Commun. 2025 Mar 19;5(3):458–76. doi: 10.1158/2767-9764.CRC-24-0261 (PMC11921867; doi:10.1158/2767-9764.CRC-24-0261)
Supplement: Supplemental Figure S3 — Chemotherapy response of TNBC cell lines [file crc-24-0261_supplemental_figure_s3_suppsf3.pdf]

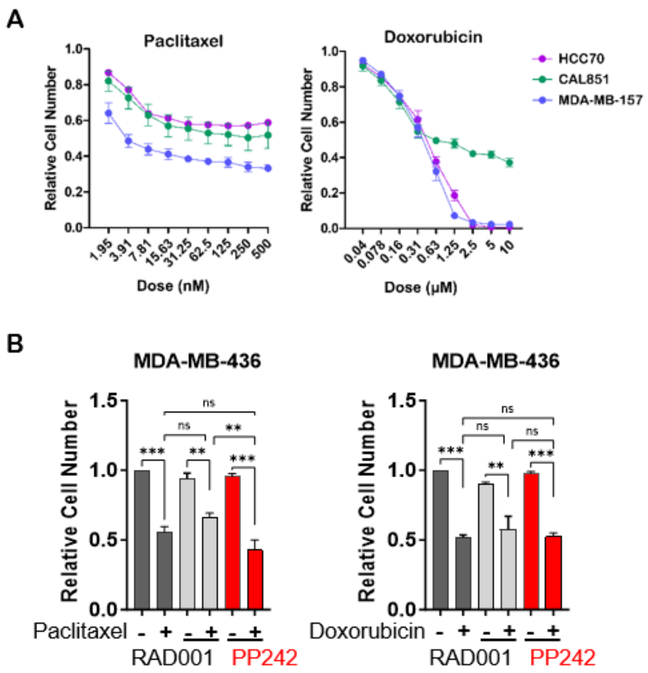

Supplemental Figure S3. Chemotherapy response of TNBC cell lines. A) TNBC cell lines were treated with a dose curve of doxorubicin and paclitaxel and cell numbers were assessed 48 hrs post treatment. B) Cell titer was assessed in MDA-MB-436 cells treated with paclitaxel or doxorubicin alone or in combination with RAD001 (200 nM) or PP242 (250 nM). P values calculated using one-way ANOVA.
