# Supplementary material for: Induction of Triple-Negative Breast Cancer Cell Death and Chemosensitivity Using mTORC2-Directed RNAi Nanomedicine
Source: Cancer Res Commun. 2025 Mar 19;5(3):458–76. doi: 10.1158/2767-9764.CRC-24-0261 (PMC11921867; doi:10.1158/2767-9764.CRC-24-0261)
Supplement: Supplemental Figure S4 — Validation of target silencing activity of siRictor and siRaptor [file crc-24-0261_supplemental_figure_s4_suppsf4.pdf]

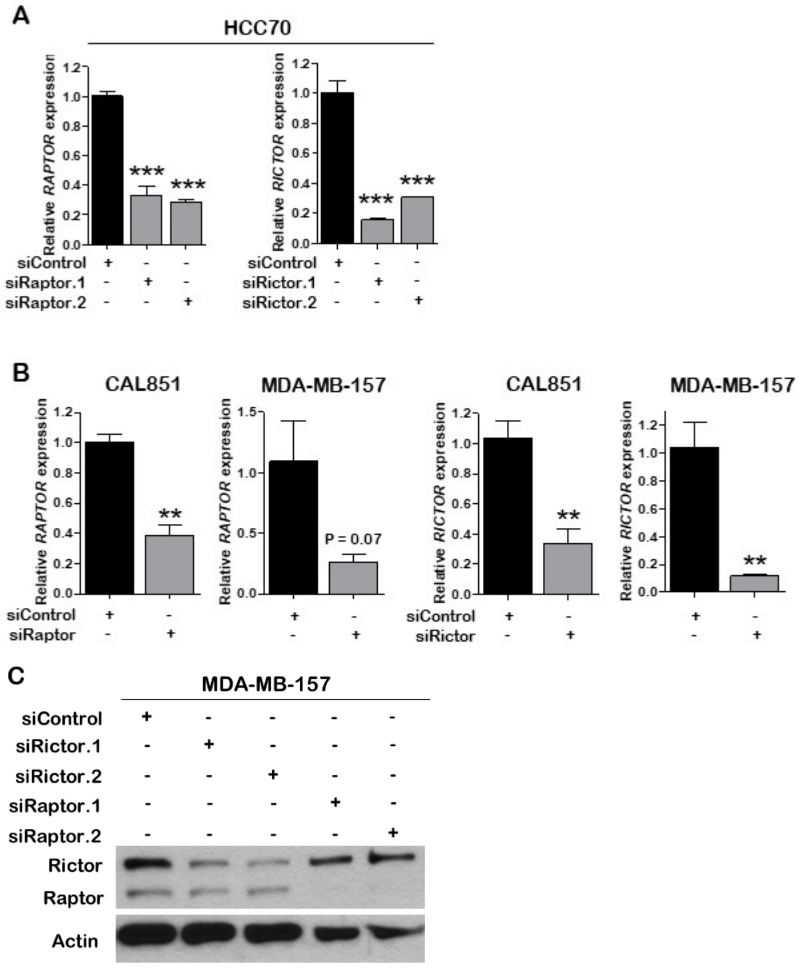

Supplemental Figure S4. Validation of target silencing activity of siRictor and siRaptor. A) mRNA knockdown of *RICTOR* and *RAPTOR* using siRNAs was confirmed in HCC70 cells by qRT-PCR. One-way ANOVA analysis with Dunnett's multiple comparison test. B) Lead siRictor and siRaptor siRNAs were also confirmed for knockdown in CAL-85-1 and MDA-MB-157 cell lines. Unpaired *t*-test. siRNAs were delivered at a 50 nM dose, and mRNA was harvested for analysis at 48 hr following treatment. C) Additional western blot analysis in MDA-MB-157 cells was used to confirm that both Rictor and Raptor siRNA sequences used achieved decrease in the downstream proteins targeted.
