# Supplementary material for: Induction of Triple-Negative Breast Cancer Cell Death and Chemosensitivity Using mTORC2-Directed RNAi Nanomedicine
Source: Cancer Res Commun. 2025 Mar 19;5(3):458–76. doi: 10.1158/2767-9764.CRC-24-0261 (PMC11921867; doi:10.1158/2767-9764.CRC-24-0261)
Supplement: Supplemental Figure S5 — Combination treatment of siRictor and chemotherapies [file crc-24-0261_supplemental_figure_s5_suppsf5.pdf]

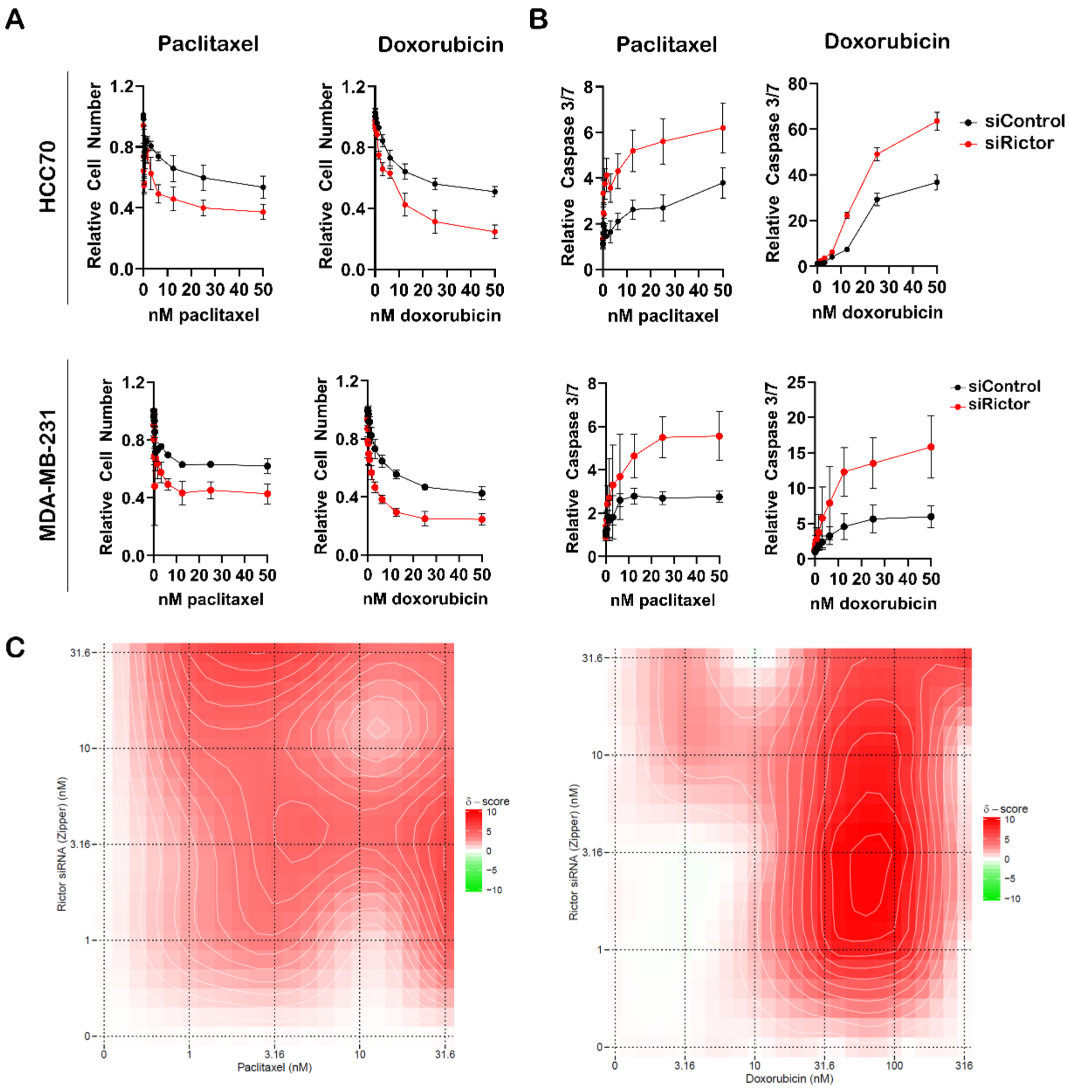

Supplemental Figure S5. Combination treatment of siRictor and chemotherapies. HCC70 and MDA-MB-231 cells were transfected with siControl or siRictor and treated with increasing doses of paclitaxel and doxorubicin. A) Cell number and B) caspase 3/7 activity were assessed at 96 hr following treatment. C) HCC70 cells were transfected with increasing doses of siRictor and increasing doses of chemotherapy. Synergy plots were generated from the viability data where red area and intensity correlates with a higher synergy score.
