# Supplementary material for: Induction of Triple-Negative Breast Cancer Cell Death and Chemosensitivity Using mTORC2-Directed RNAi Nanomedicine
Source: Cancer Res Commun. 2025 Mar 19;5(3):458–76. doi: 10.1158/2767-9764.CRC-24-0261 (PMC11921867; doi:10.1158/2767-9764.CRC-24-0261)
Supplement: Supplemental Figure S6 — Development and cell growth testing of siMTOR [file crc-24-0261_supplemental_figure_s6_suppsf6.pdf]

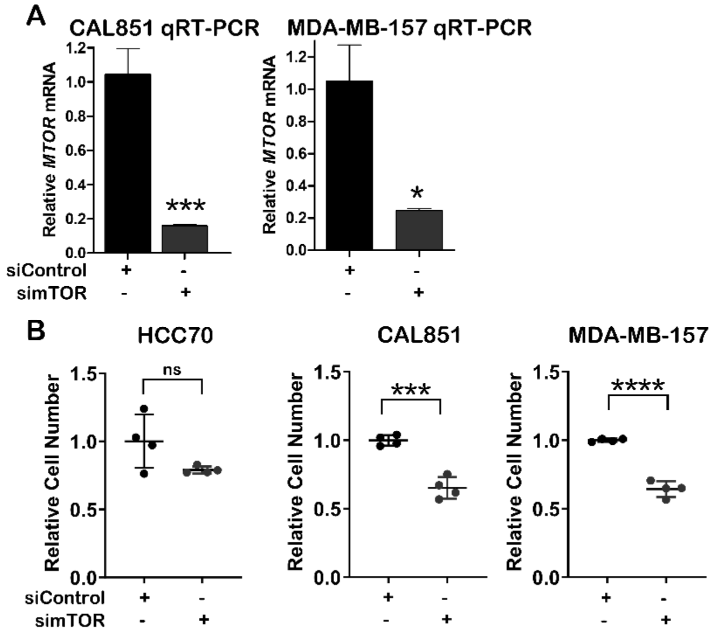

Supplemental Figure S6. Development and cell growth testing of siMTOR. A) qRT-PCR was performed in CAL-85-1 and MDA-MB-157 cell lines to confirm mRNA level knockdown achieved by siMTOR. siRNA was delivered at a 50 nM dose and mRNA was harvested for analysis at 48 hr following treatment. B) simTOR effect on cell growth was assessed by CellTiter Glo assay at 96 hr following treatment. Unpaired *t*-test.
