# Supplementary material for: Induction of Triple-Negative Breast Cancer Cell Death and Chemosensitivity Using mTORC2-Directed RNAi Nanomedicine
Source: Cancer Res Commun. 2025 Mar 19;5(3):458–76. doi: 10.1158/2767-9764.CRC-24-0261 (PMC11921867; doi:10.1158/2767-9764.CRC-24-0261)
Supplement: Supplemental Figure S7 — Phospho-kinase array [file crc-24-0261_supplemental_figure_s7_suppsf7.pdf]

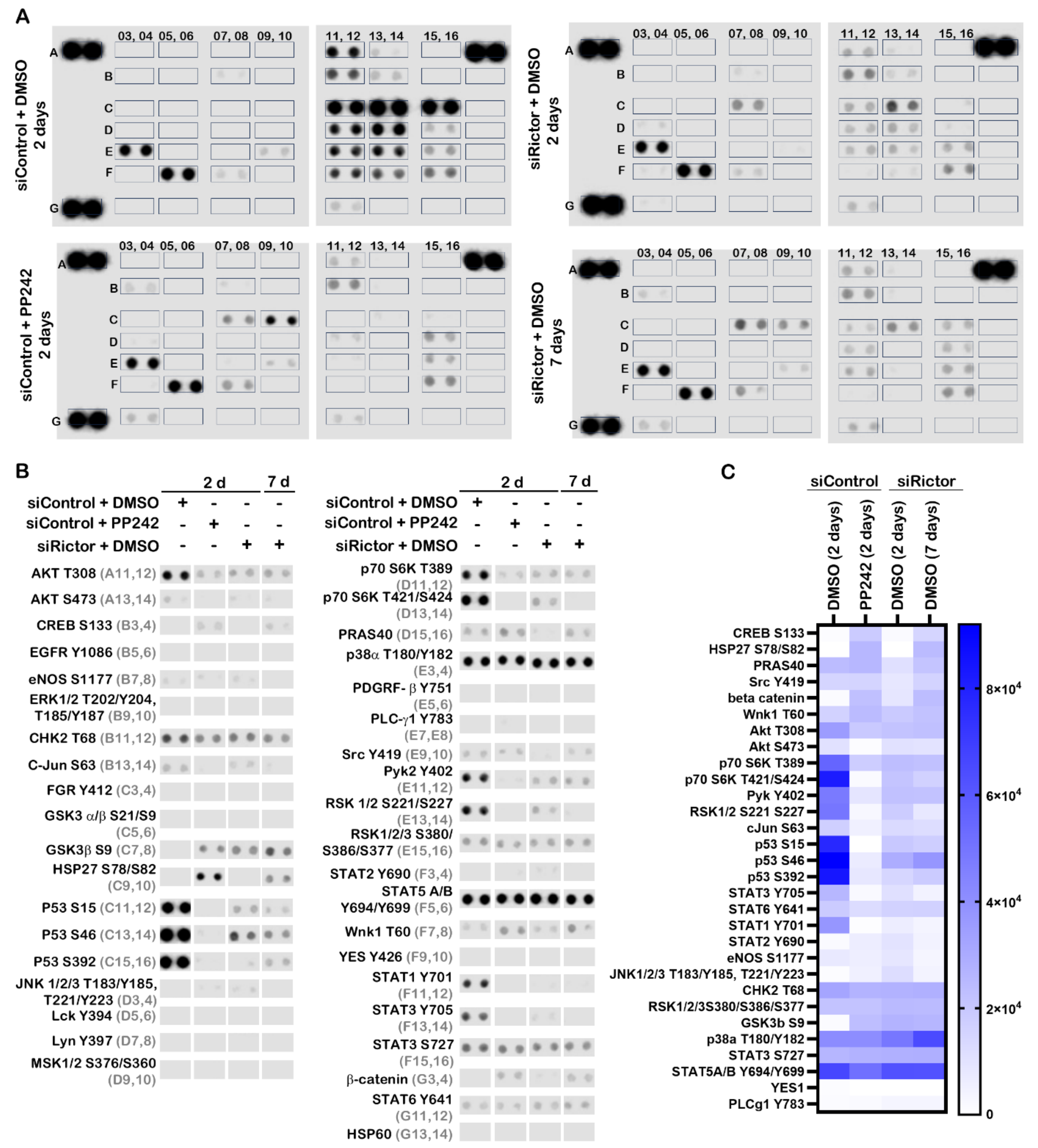

Supplemental Figure S7. Phospho-kinase array. A) HCC70 cells treated with either PP242 or siRictor were assessed by protein array. A full array membrane for each treatment is shown, with coordinates boxing each target within the array. B) Treatments are shown organized by target. Each target is indicated on the left, and the matching array coordinates for that target are indicated inside gray parentheses. C) Protein quantitation was measured by densitometry. Values are shown as average ± SD N = 3. One-way ANOVA.
