# Supplementary material for: Induction of Triple-Negative Breast Cancer Cell Death and Chemosensitivity Using mTORC2-Directed RNAi Nanomedicine
Source: Cancer Res Commun. 2025 Mar 19;5(3):458–76. doi: 10.1158/2767-9764.CRC-24-0261 (PMC11921867; doi:10.1158/2767-9764.CRC-24-0261)
Supplement: Supplemental Figure S8 — Tumor cell uptake of si-NPs [file crc-24-0261_supplemental_figure_s8_suppsf8.pdf]

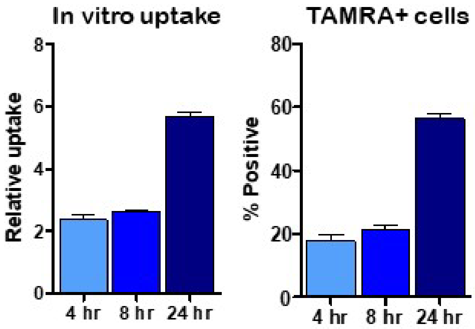

Supplemental Figure S8. Tumor cell uptake of si-NPs. si-NP uptake was quantified in HCC70 cells over time by flow cytometric detection of the fluorescent TAMRA-tagged siRNA.
