# Supplementary material for: Induction of Triple-Negative Breast Cancer Cell Death and Chemosensitivity Using mTORC2-Directed RNAi Nanomedicine
Source: Cancer Res Commun. 2025 Mar 19;5(3):458–76. doi: 10.1158/2767-9764.CRC-24-0261 (PMC11921867; doi:10.1158/2767-9764.CRC-24-0261)
Supplement: Supplemental Figure S9 — siRictor-NPs on target silencing and induction of apoptosis in TNBCs [file crc-24-0261_supplemental_figure_s9_suppsf9.pdf]

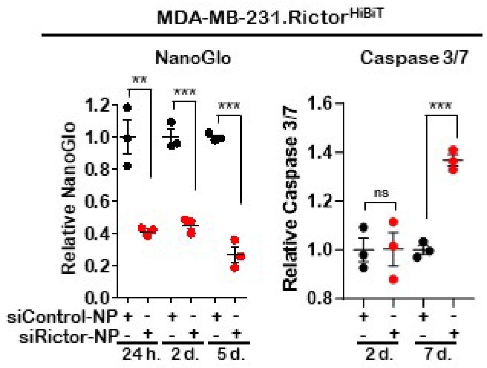

Supplemental Figure S9. siRictor-NPs on target silencing and induction of apoptosis in TNBCs. MDA-MB-231 Rictor^{HiBiT} cells were treated with 100 nM siRictor-NPs, and Rictor silencing was quantified at multiple days post treatment by HiBiT peptide bioluminescence generated from split Nano-Luciferase complementation. MDA-MB-231 Rictor^{HiBiT} cell apoptosis was also quantified following siRictor-NP treatment. Unpaired *t*-test.
