# Supplementary material for: Induction of Triple-Negative Breast Cancer Cell Death and Chemosensitivity Using mTORC2-Directed RNAi Nanomedicine
Source: Cancer Res Commun. 2025 Mar 19;5(3):458–76. doi: 10.1158/2767-9764.CRC-24-0261 (PMC11921867; doi:10.1158/2767-9764.CRC-24-0261)
Supplement: Supplemental Figure S10 — siRictor-NP mediated mRNA knockdown [file crc-24-0261_supplemental_figure_s10_suppsf10.pdf]

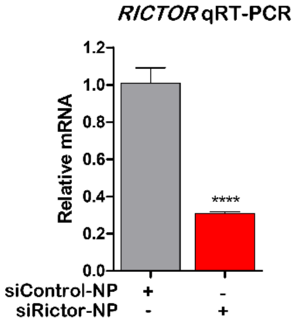

Supplemental Figure S10. siRictor-NP mediated mRNA knockdown. HCC70 cells were treated with 50 nM siRictor-NPs and *RICTOR* mRNA silencing was confirmed by qRT-PCR 72 hours following treatment. Unpaired *t*-test.
