# Supplementary material for: Induction of Triple-Negative Breast Cancer Cell Death and Chemosensitivity Using mTORC2-Directed RNAi Nanomedicine
Source: Cancer Res Commun. 2025 Mar 19;5(3):458–76. doi: 10.1158/2767-9764.CRC-24-0261 (PMC11921867; doi:10.1158/2767-9764.CRC-24-0261)
Supplement: Supplemental Figure S11 — siRictor-NP treatment impact on cell proliferation [file crc-24-0261_supplemental_figure_s11_suppsf11.pdf]

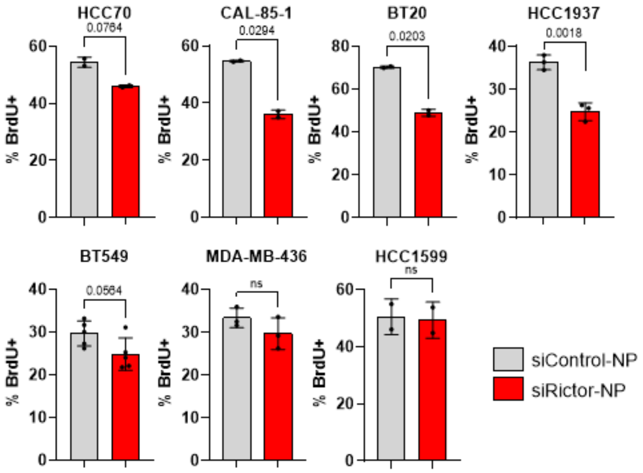

Supplemental Figure S11. siRictor-NP treatment impact on cell proliferation. Cells were treated with 200 nM siRictor-NPs and quantified for BrdU incorporation as a measure of cell proliferation. Unpaired *t*-test.
