# Supplementary material for: Induction of Triple-Negative Breast Cancer Cell Death and Chemosensitivity Using mTORC2-Directed RNAi Nanomedicine
Source: Cancer Res Commun. 2025 Mar 19;5(3):458–76. doi: 10.1158/2767-9764.CRC-24-0261 (PMC11921867; doi:10.1158/2767-9764.CRC-24-0261)
Supplement: Supplemental Figure S13 — Extended tumor data for short-term siRictor-NP therapeutic effects in HCC70 tumor-bearing mice [file crc-24-0261_supplemental_figure_s13_suppsf13.pdf]

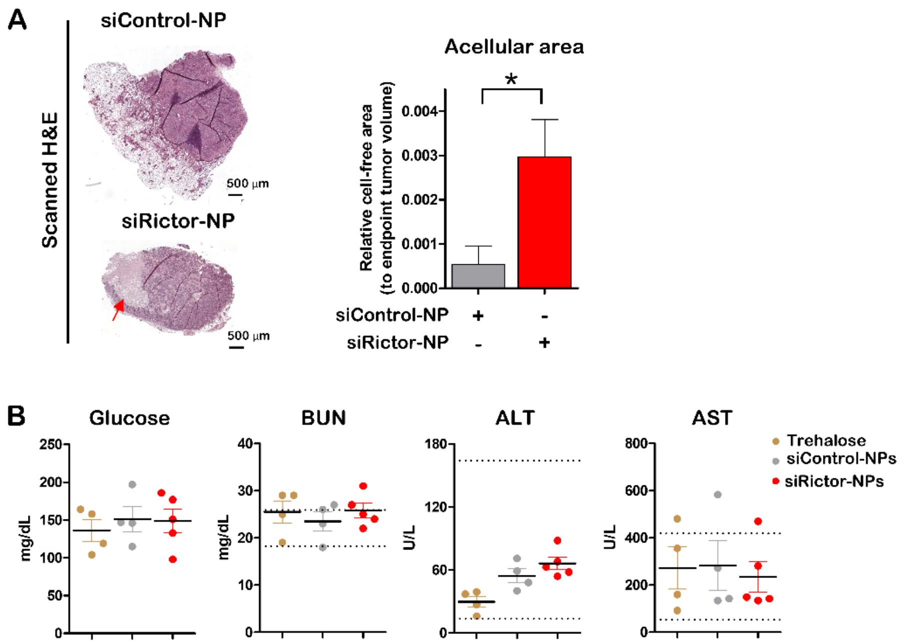

Supplemental Figure S13. Extended tumor data for short-term siRictor-NP therapeutic effects in HCC70 tumor-bearing mice. A) Scanned H&E images of siControl-NP and siRictor-NP tumors were quantified for areas of acellularity. B) Mouse plasma collected on Day 7 was assessed for changes in glucose levels following si-NP treatment, as well as elevation in kidney and liver damage markers.
