# Supplementary material for: Induction of Triple-Negative Breast Cancer Cell Death and Chemosensitivity Using mTORC2-Directed RNAi Nanomedicine
Source: Cancer Res Commun. 2025 Mar 19;5(3):458–76. doi: 10.1158/2767-9764.CRC-24-0261 (PMC11921867; doi:10.1158/2767-9764.CRC-24-0261)
Supplement: Supplemental Figure S14 — siRictor-NPs combination with chemotherapy [file crc-24-0261_supplemental_figure_s14_suppsf14.pdf]

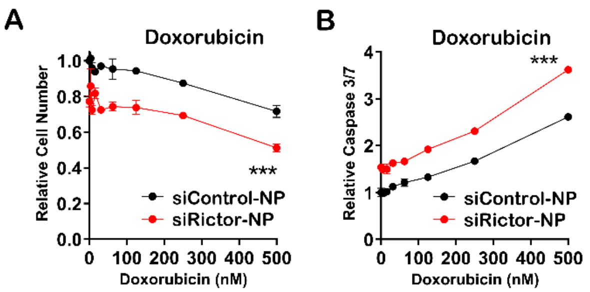

Supplemental Figure S14. siRictor-NPs combination with chemotherapy. HCC70 cells were treated with 100 nM si-NPs ± doxorubicin in increasing doses. Cell viability was measured by CellTiter Glo 2 days after chemotherapy addition (A), and Caspase 3/7 activity was measured by Caspase Glo 1 day after chemotherapy addition (B). Statistical significance was calculated based on area under the curve analysis. Unpaired *t*-test.
