# Supplementary material for: Induction of Triple-Negative Breast Cancer Cell Death and Chemosensitivity Using mTORC2-Directed RNAi Nanomedicine
Source: Cancer Res Commun. 2025 Mar 19;5(3):458–76. doi: 10.1158/2767-9764.CRC-24-0261 (PMC11921867; doi:10.1158/2767-9764.CRC-24-0261)
Supplement: Supplemental Figure S15 — Extended tumor data for siRictor-NP combination with paclitaxel in HCC70 tumor-bearing mice [file crc-24-0261_supplemental_figure_s15_suppsf15.pdf]

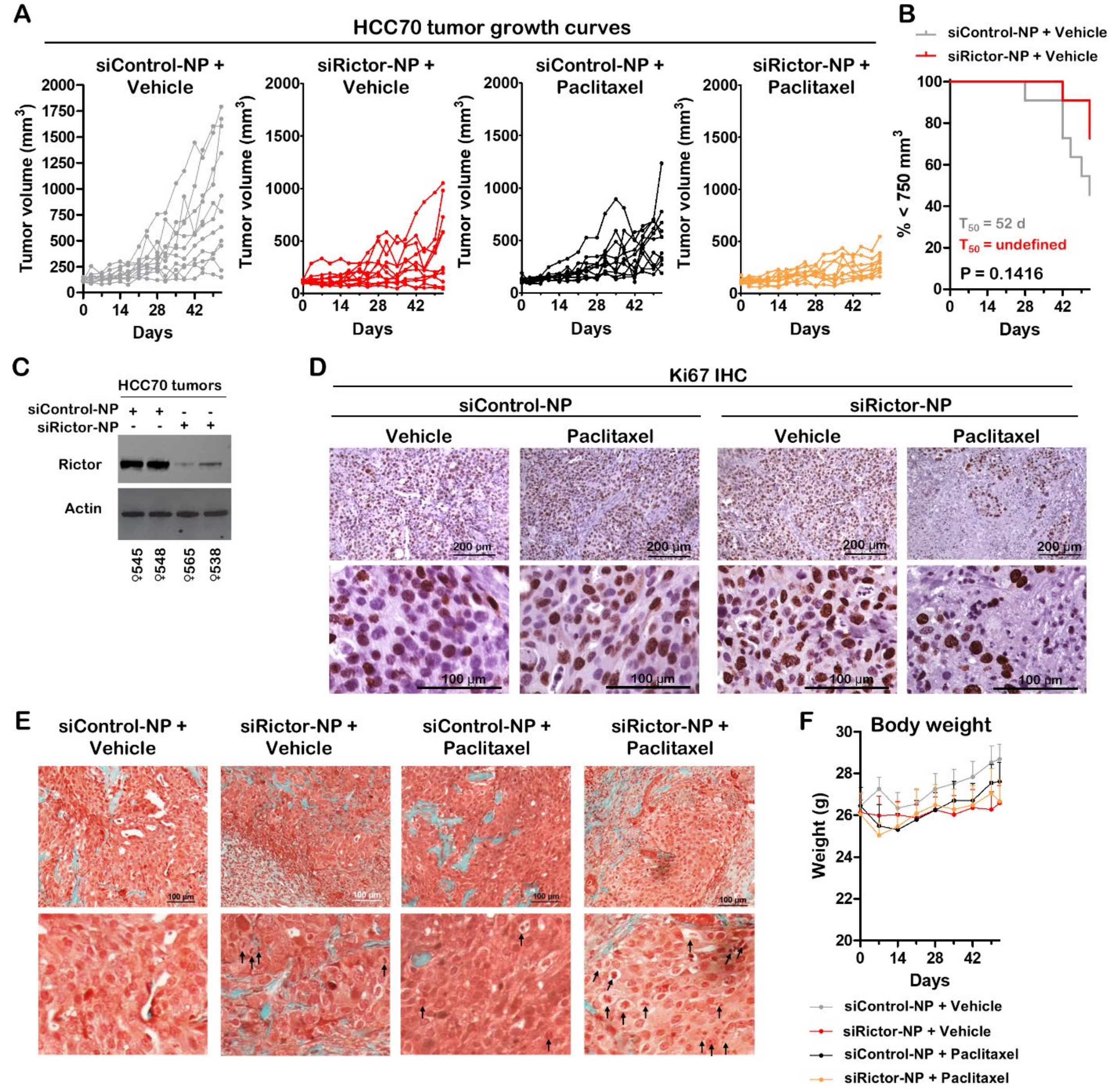

Supplemental Figure S15. Extended tumor data for siRictor-NP combination with paclitaxel in HCC70 tumor-bearing mice. A) Tumor growth curves for individual mice in each treatment group are shown. B) Kaplan-Meier analysis of tumor-bearing mice, defining survival as tumor volume under 750 mm^3 (siControl-NP treated mice). C) Tumor lysates were probed for Rictor knockdown by western blot analysis. D) Representative Ki67 IHC staining of treated tumors at study endpoint. E) Representative trichrome staining of treated tumors at study endpoint. Arrows indicate cells with mitotic figures or cells dying in mitosis. F) Mouse body weight was monitored throughout the study.
